# Supplementary figures and images for: mTORC1 senses glutamine and other amino acids through GCN2
Source: EMBO J. 2025 Jul 21;44(17):4825–66. doi: 10.1038/s44318-025-00505-1 (PMC12402317; doi:10.1038/s44318-025-00505-1)

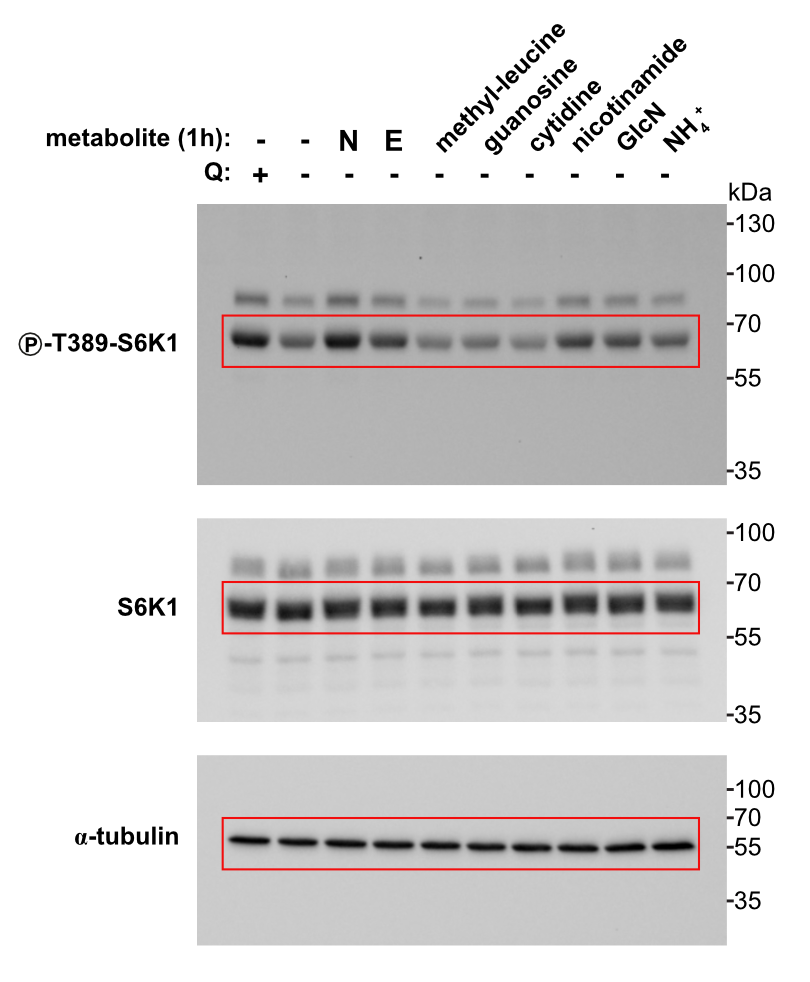

Supplement: Supplementary file 4 — Source data Fig. 1 [file 44318_2025_505_MOESM4_ESM.zip › Figure 1/1B-C/uncropped blots.tiff]

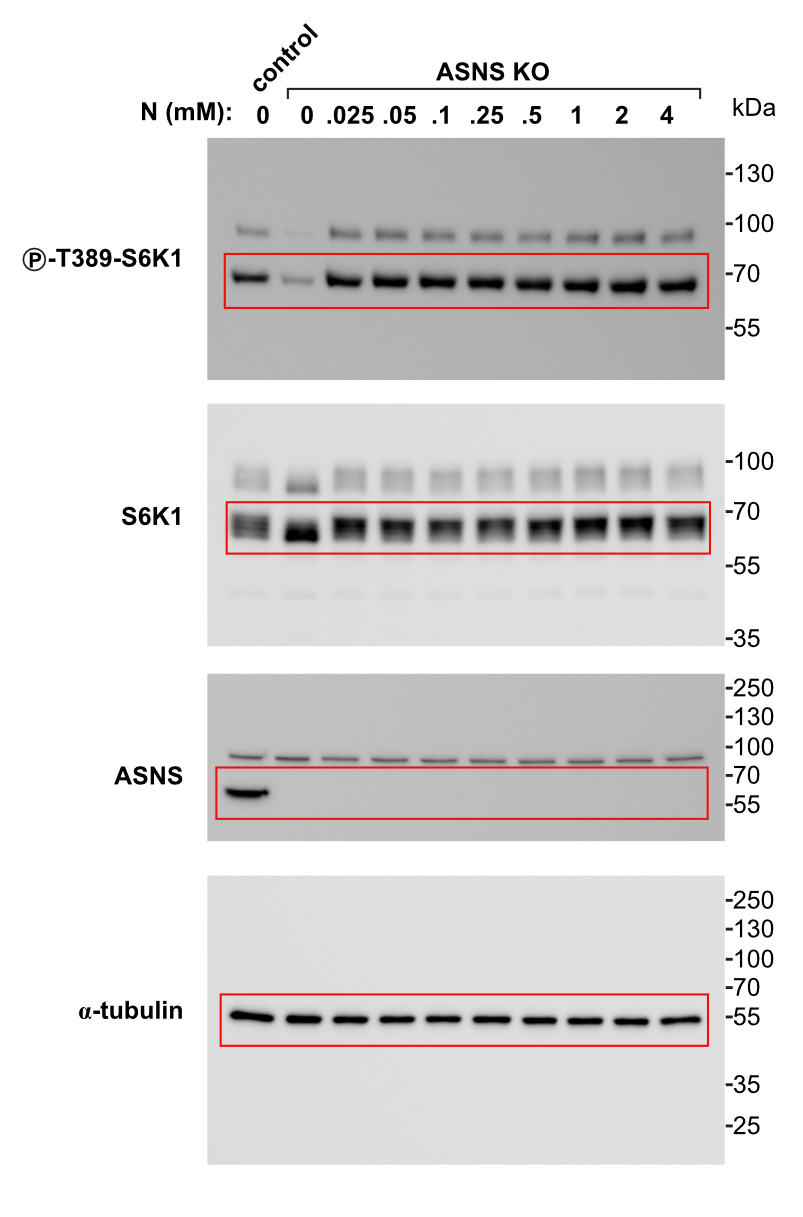

Supplement: Supplementary file 4 — Source data Fig. 1 [file 44318_2025_505_MOESM4_ESM.zip › Figure 1/1G-H/uncropped blots.tiff]

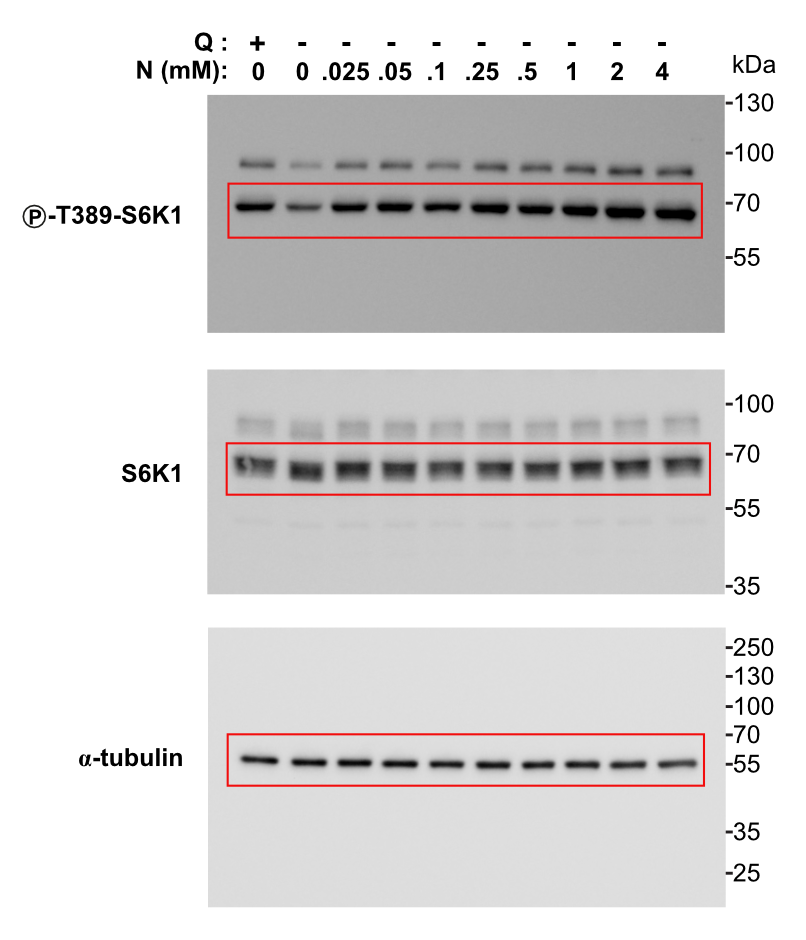

Supplement: Supplementary file 4 — Source data Fig. 1 [file 44318_2025_505_MOESM4_ESM.zip › Figure 1/1D-E/uncropped blots.tiff]

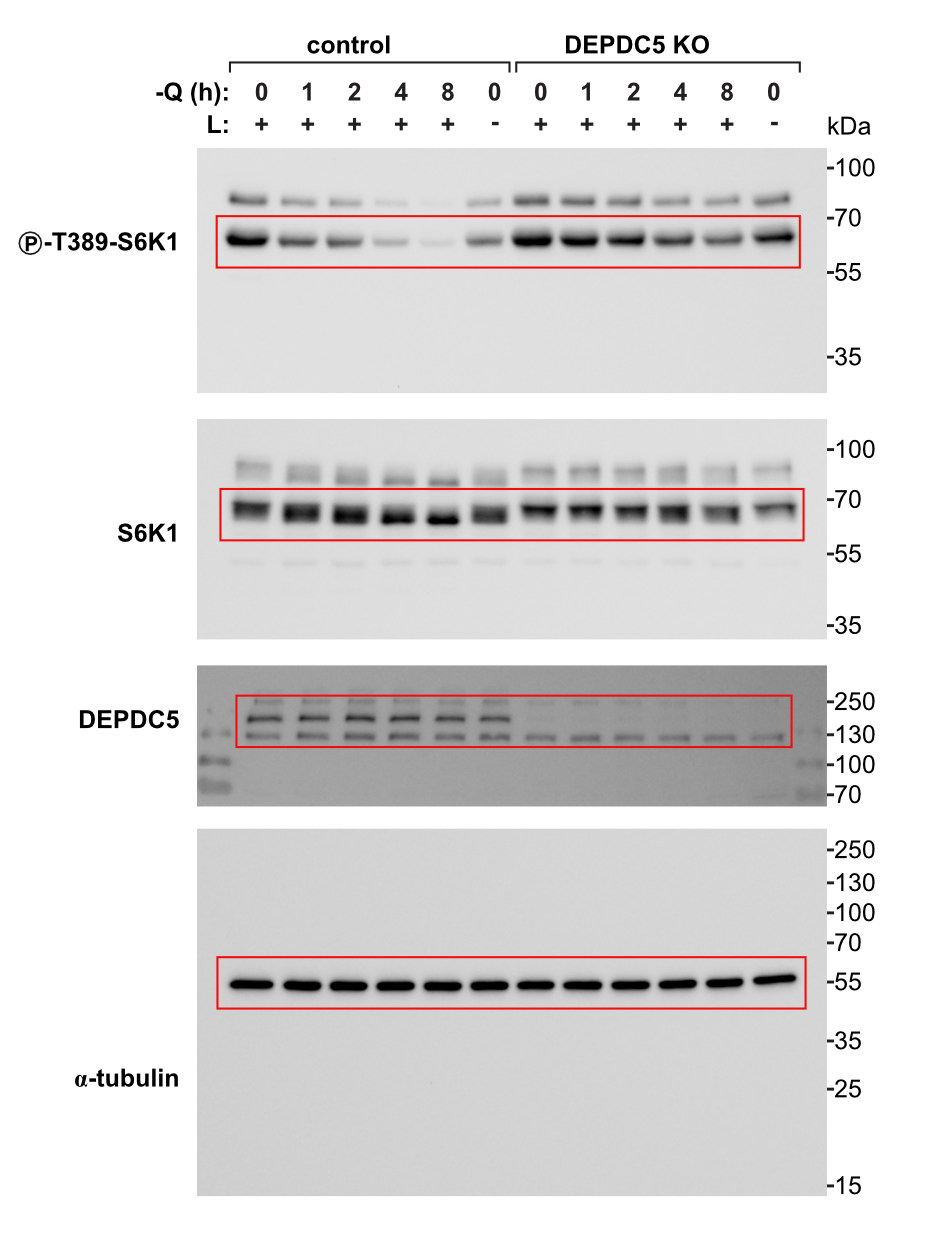

Supplement: Supplementary file 5 — Source data Fig. 2 [file 44318_2025_505_MOESM5_ESM.zip › Figure 2/2C-D/uncropped blots.tiff]

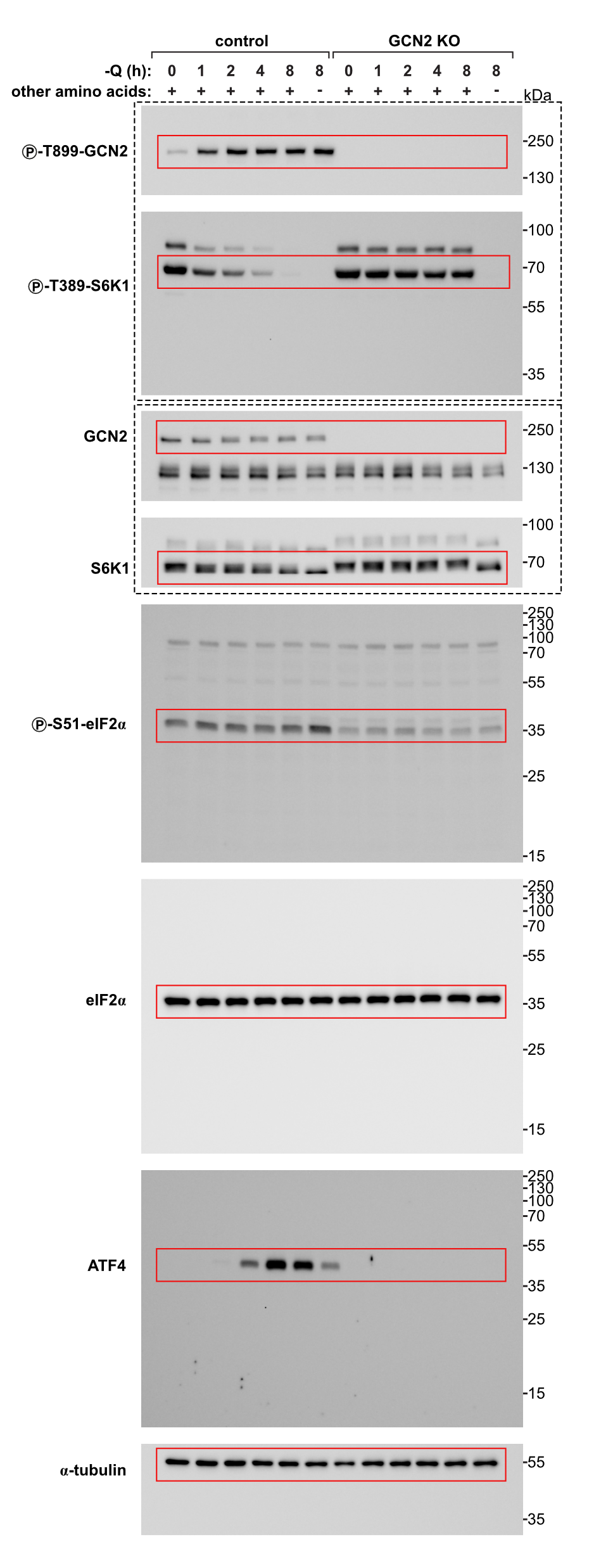

Supplement: Supplementary file 5 — Source data Fig. 2 [file 44318_2025_505_MOESM5_ESM.zip › Figure 2/2E-F/uncropped blots.tiff]

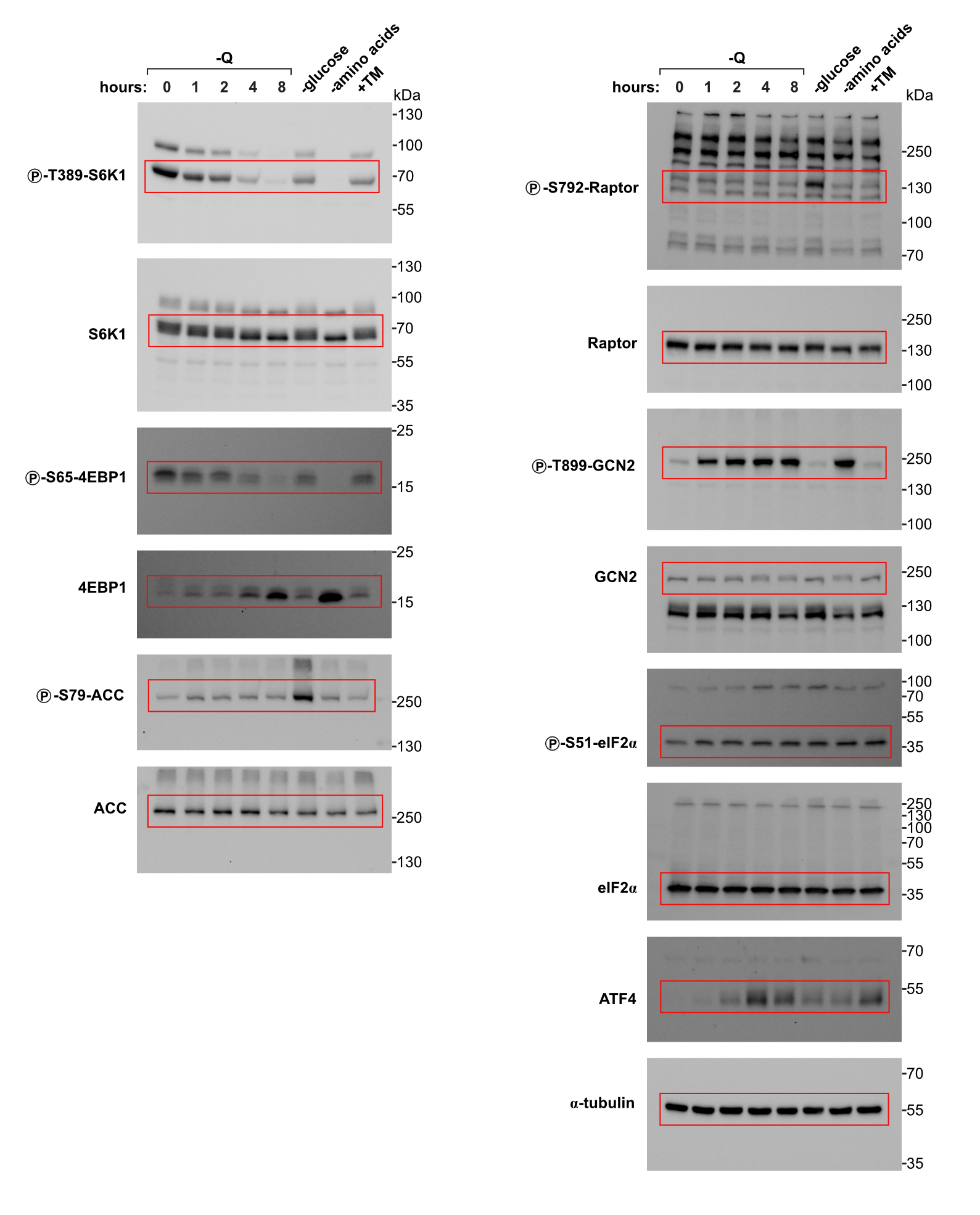

Supplement: Supplementary file 5 — Source data Fig. 2 [file 44318_2025_505_MOESM5_ESM.zip › Figure 2/2A-B/uncropped blots.tiff]

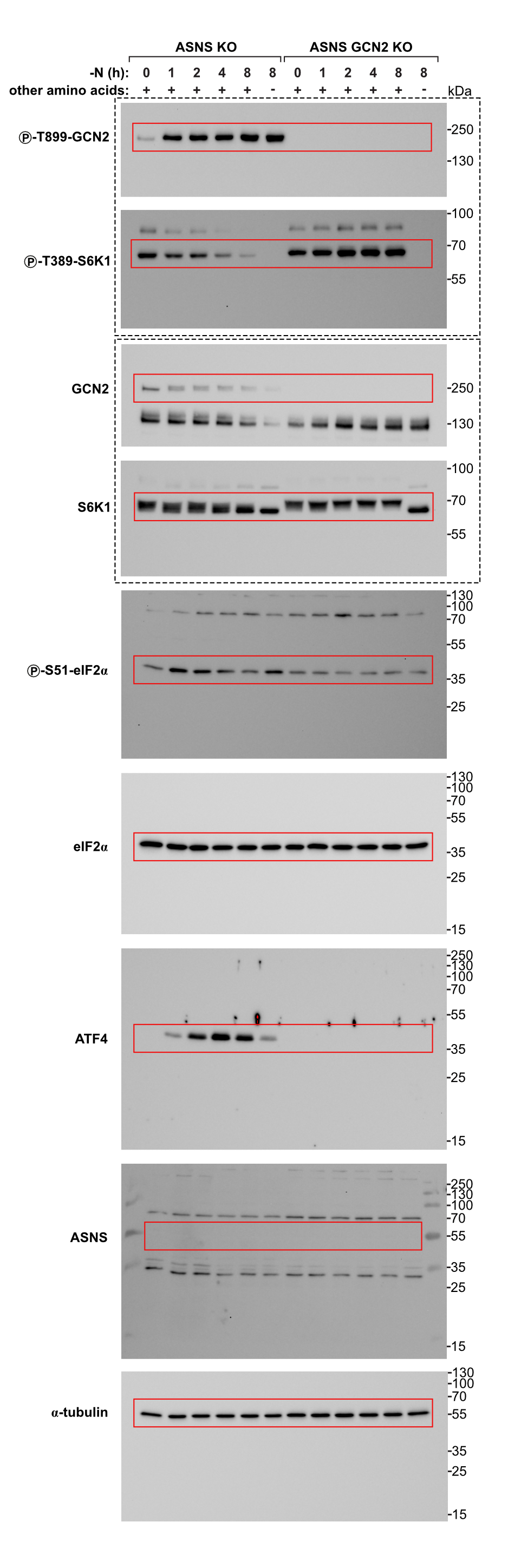

Supplement: Supplementary file 5 — Source data Fig. 2 [file 44318_2025_505_MOESM5_ESM.zip › Figure 2/2G-H/uncropped blots.tiff]

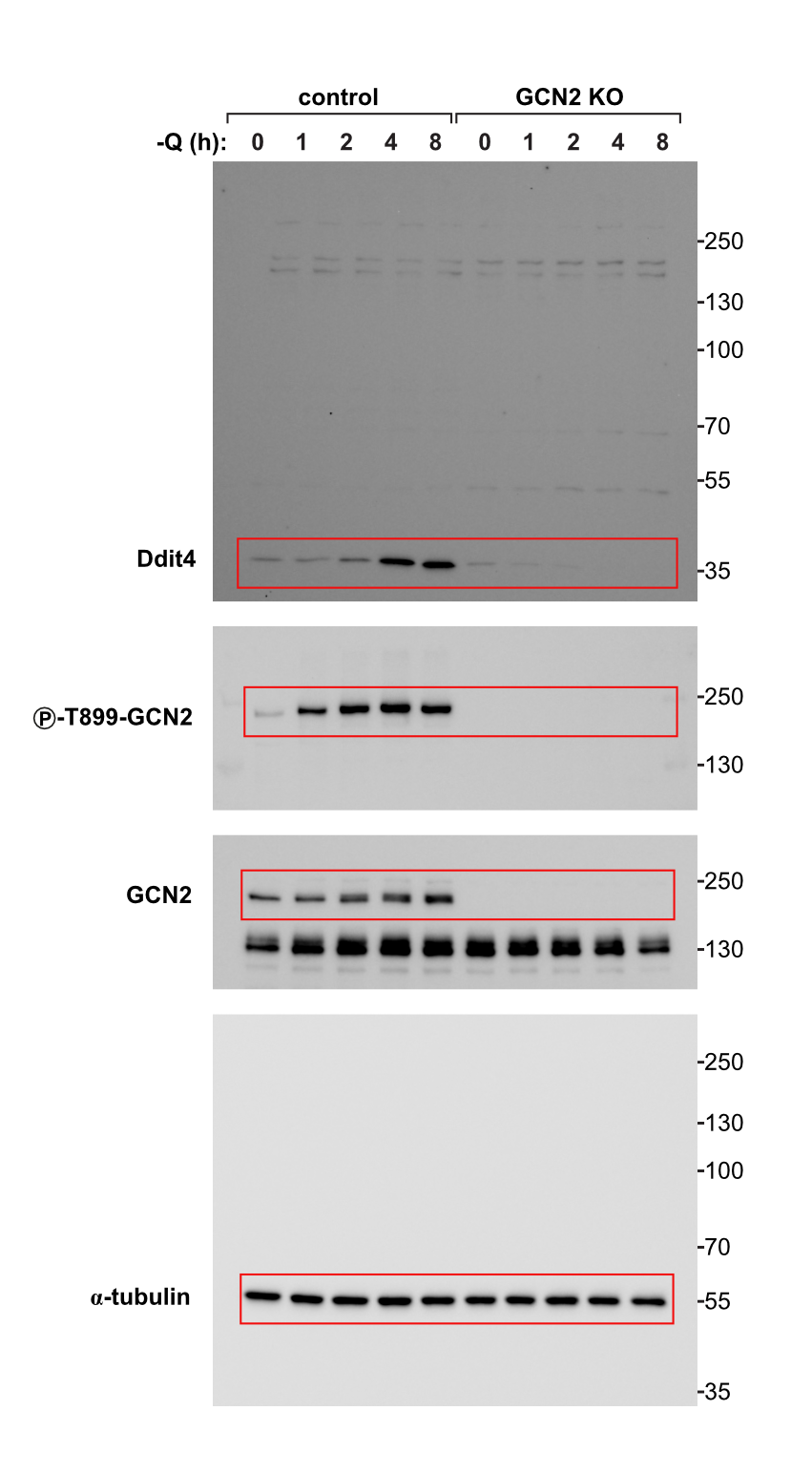

Supplement: Supplementary file 6 — Source data Fig. 3 [file 44318_2025_505_MOESM6_ESM.zip › Figure 3/3C/uncropped blots.tiff]

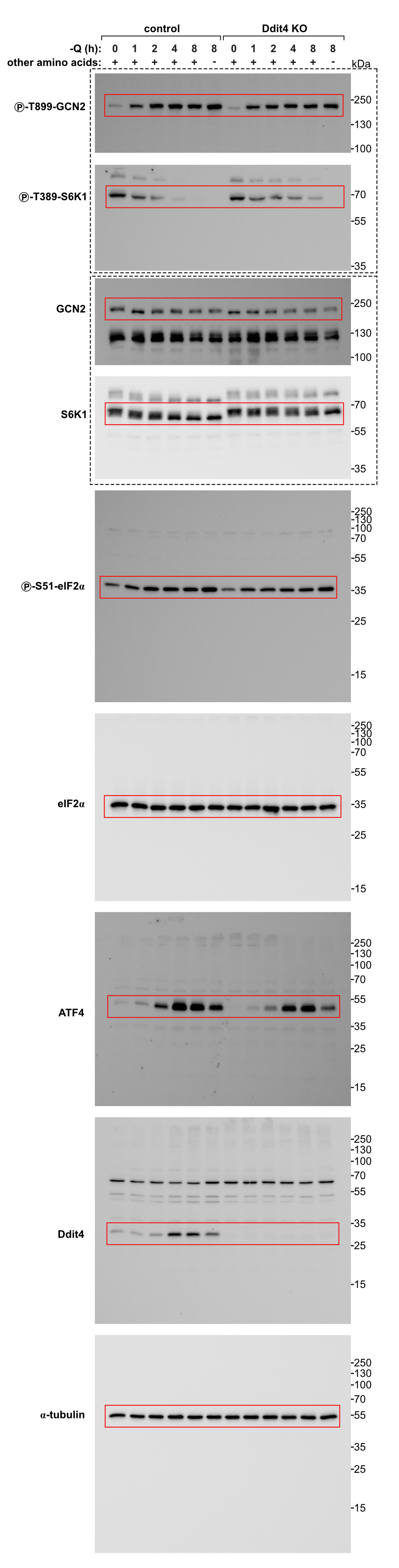

Supplement: Supplementary file 6 — Source data Fig. 3 [file 44318_2025_505_MOESM6_ESM.zip › Figure 3/3D-E/uncropped blots.tiff]

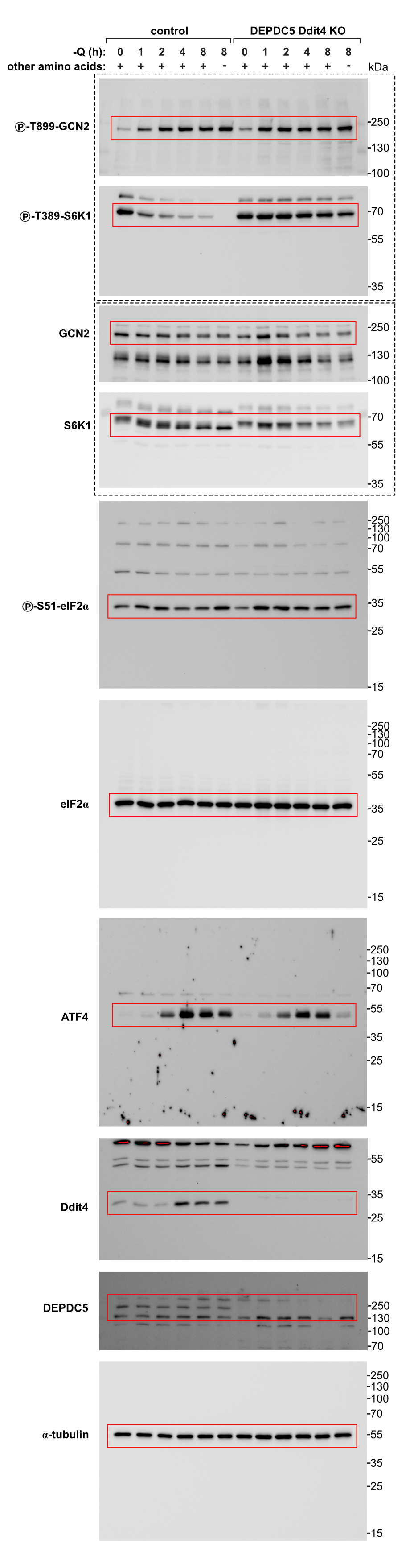

Supplement: Supplementary file 6 — Source data Fig. 3 [file 44318_2025_505_MOESM6_ESM.zip › Figure 3/3F-G/uncropped blots.tiff]

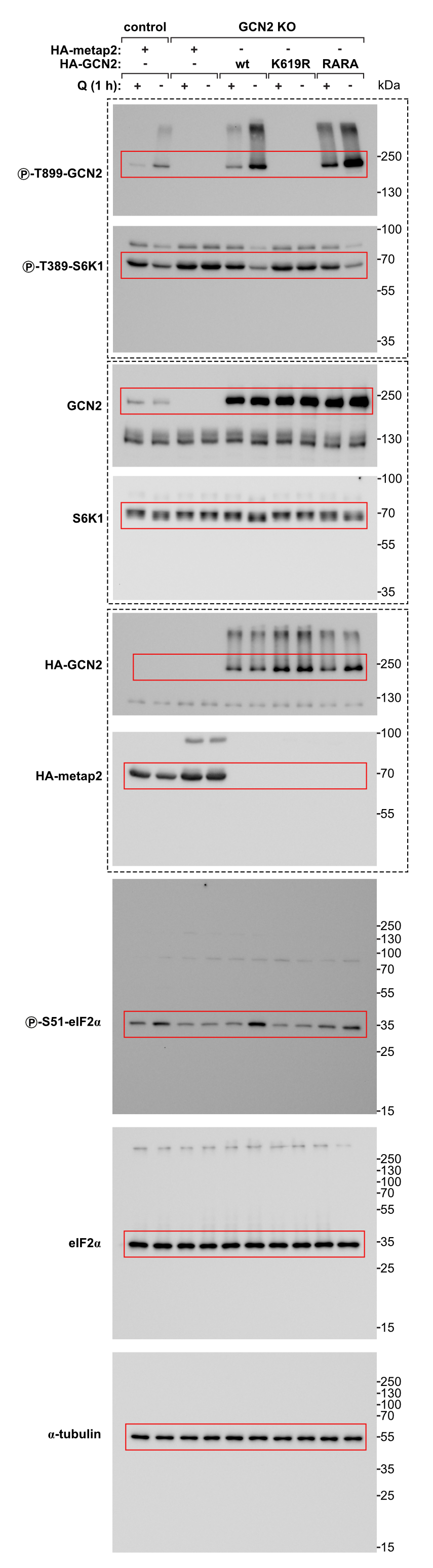

Supplement: Supplementary file 7 — Source data Fig. 4 [file 44318_2025_505_MOESM7_ESM.zip › Figure 4/4E/uncropped blots.tiff]

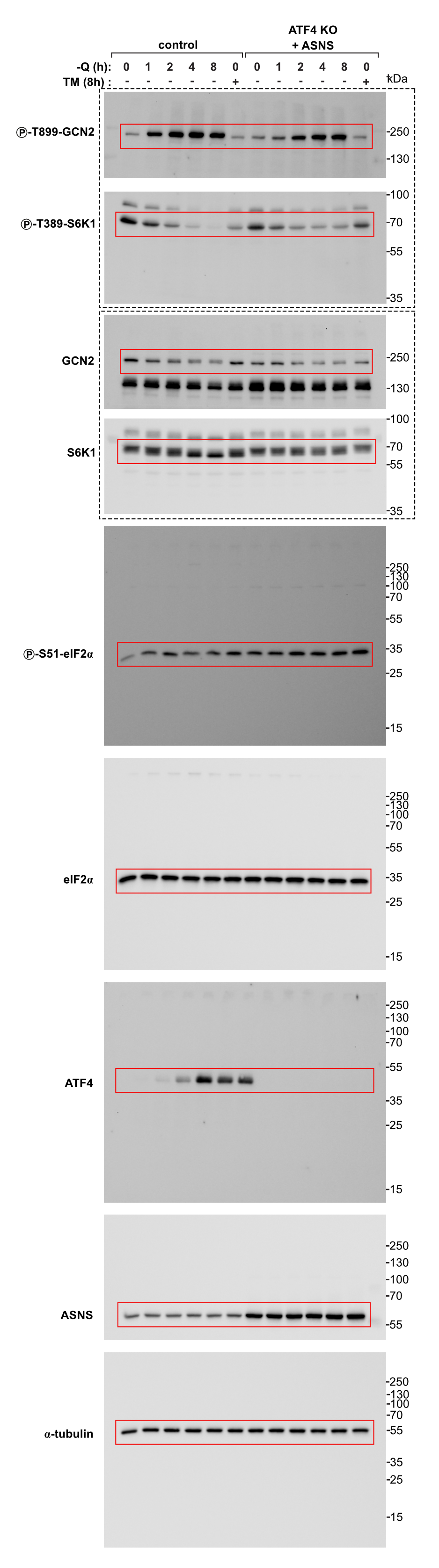

Supplement: Supplementary file 7 — Source data Fig. 4 [file 44318_2025_505_MOESM7_ESM.zip › Figure 4/4A-B/uncropped blots.tiff]

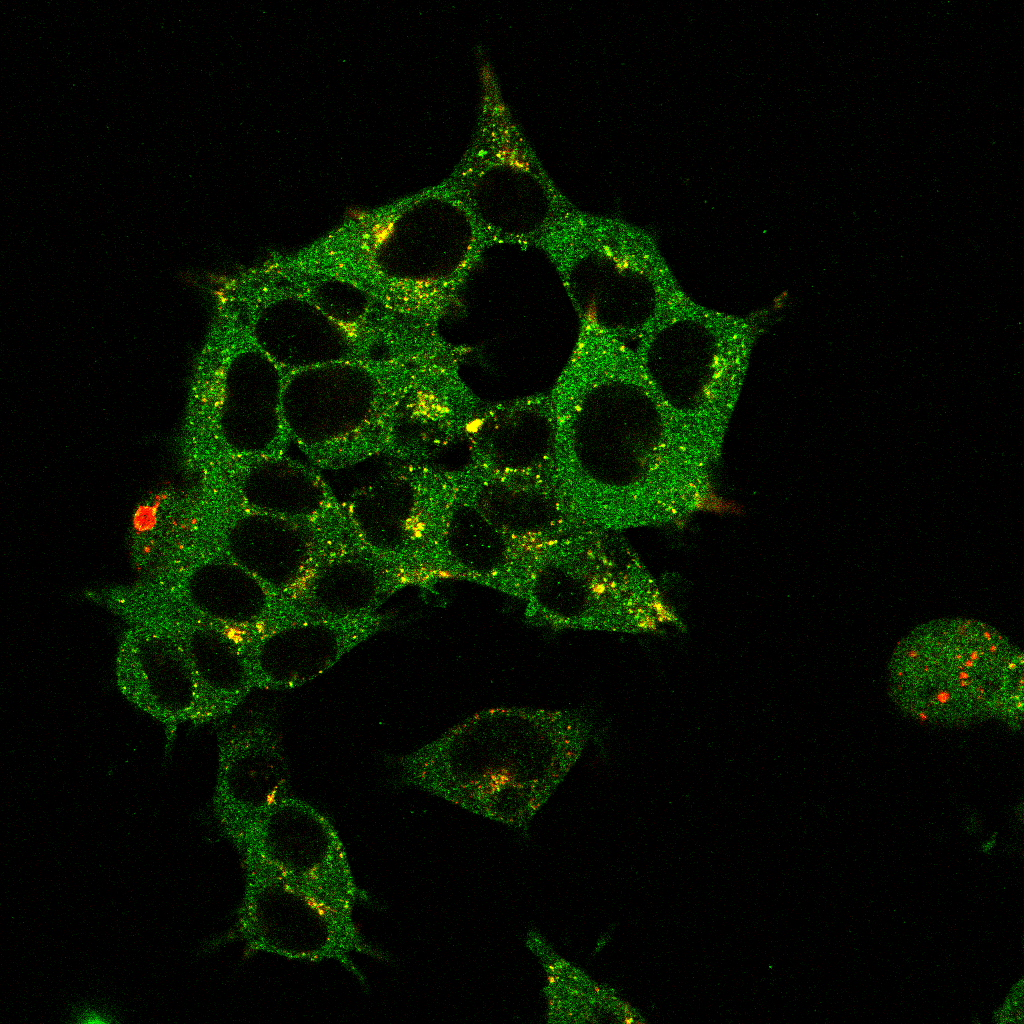

Supplement: Supplementary file 7 — Source data Fig. 4 [file 44318_2025_505_MOESM7_ESM.zip › Figure 4/4F-G/GCN2KO -Q.tif]

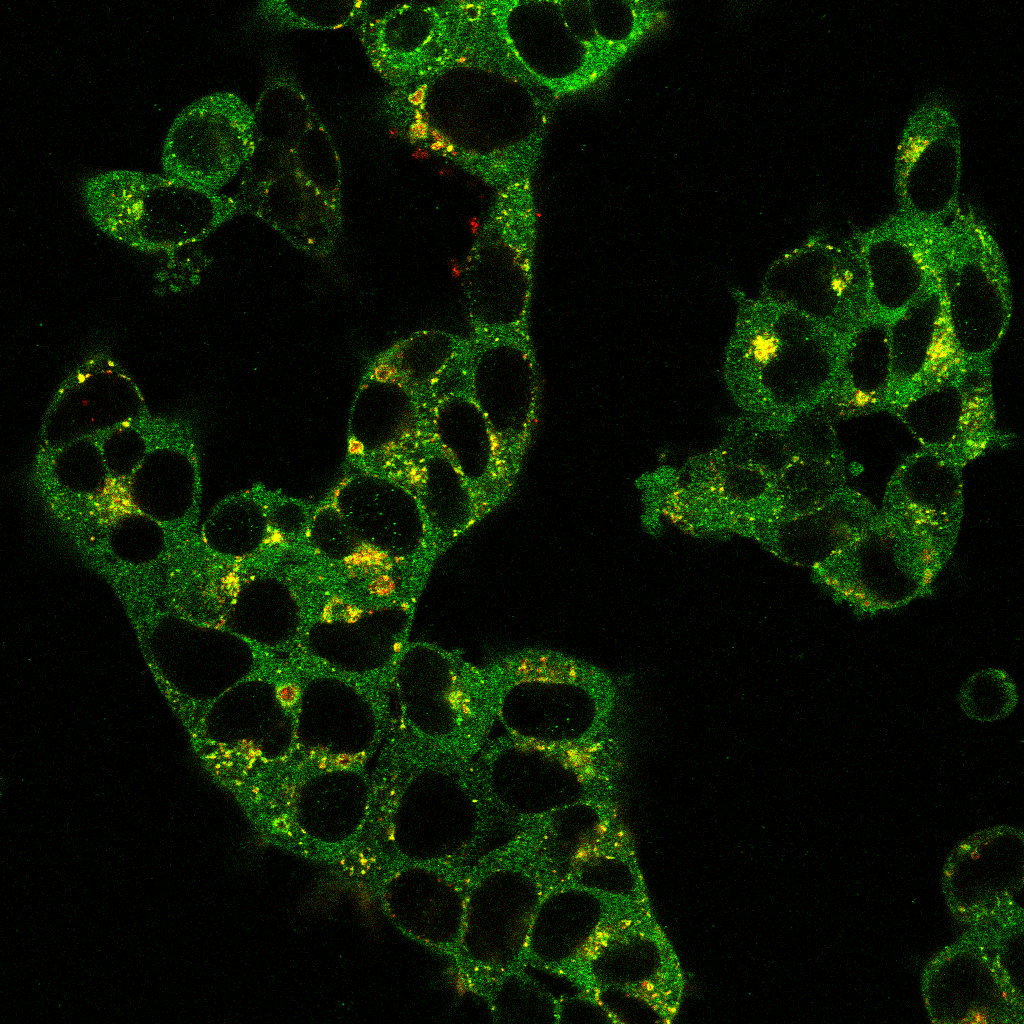

Supplement: Supplementary file 7 — Source data Fig. 4 [file 44318_2025_505_MOESM7_ESM.zip › Figure 4/4F-G/control +Q.tif]

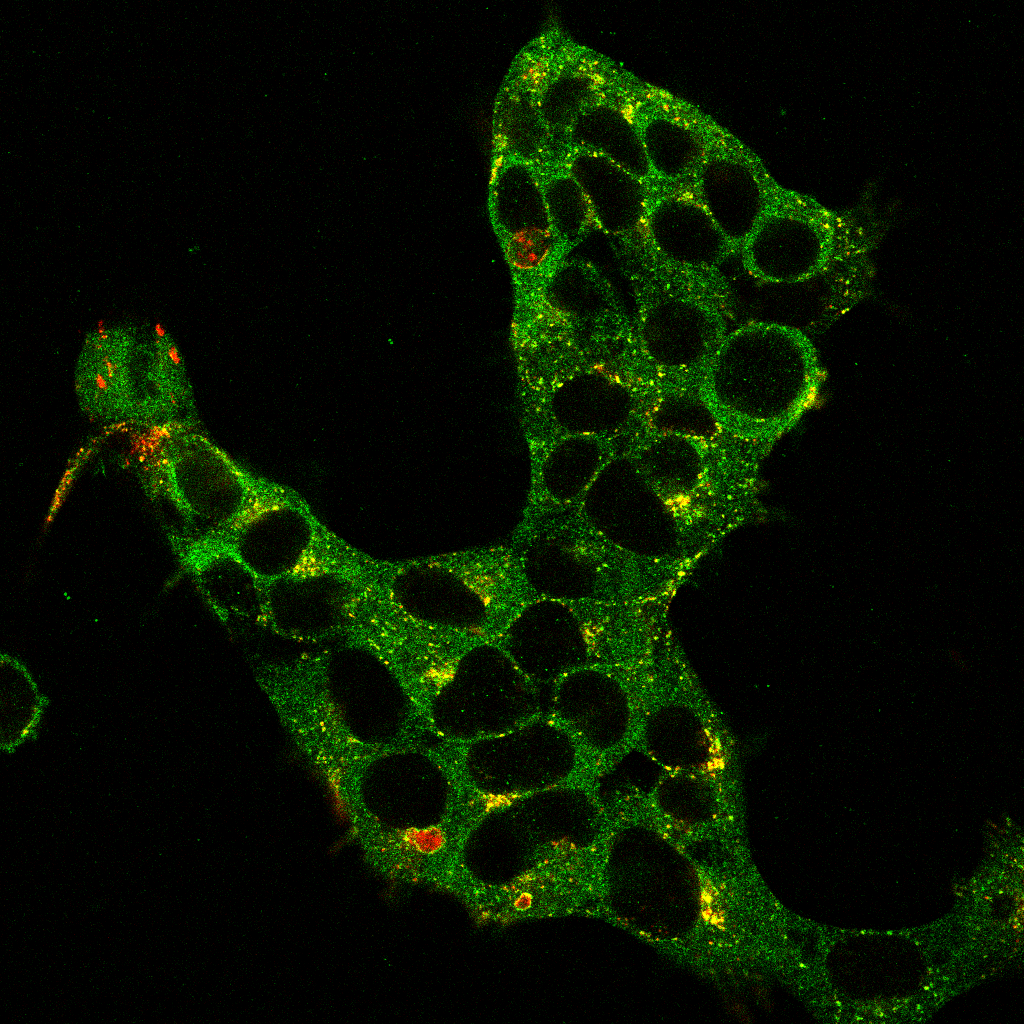

Supplement: Supplementary file 7 — Source data Fig. 4 [file 44318_2025_505_MOESM7_ESM.zip › Figure 4/4F-G/GCN2KO +Q.tif]

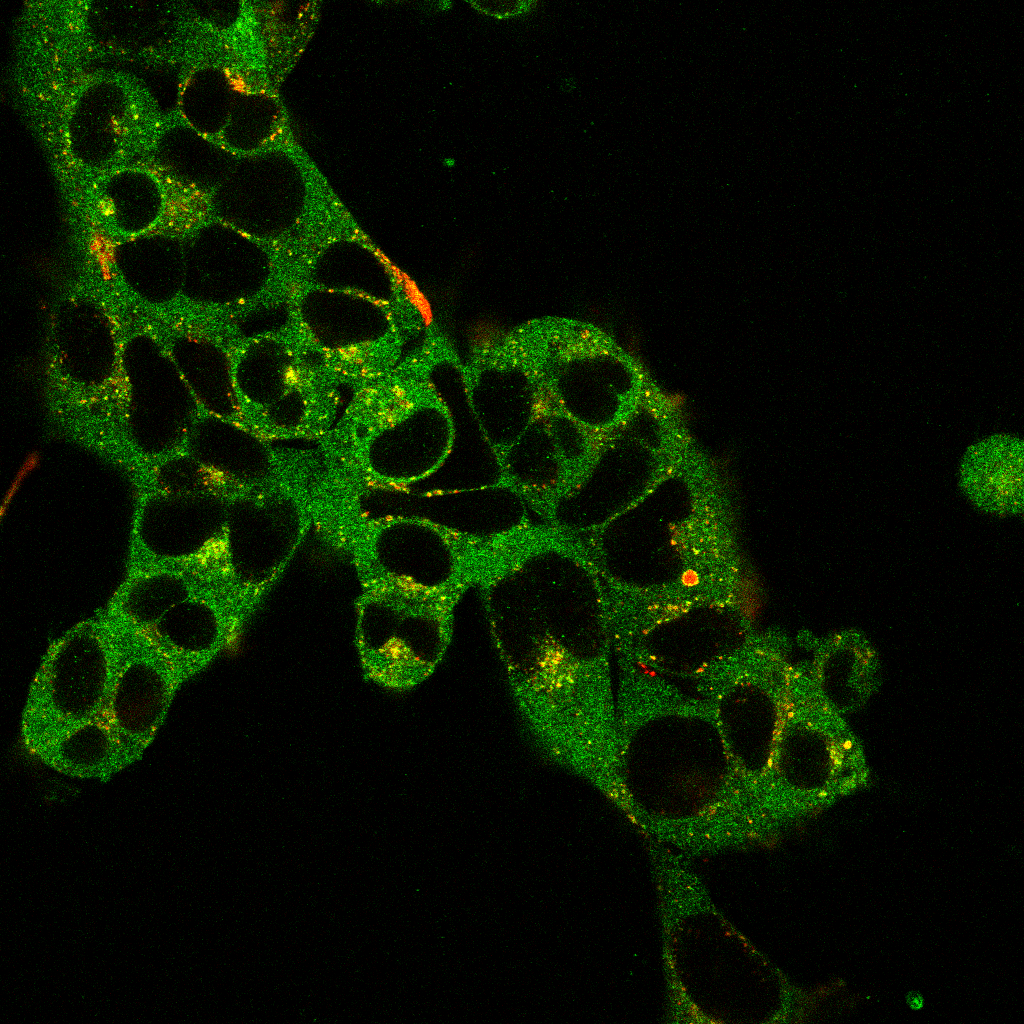

Supplement: Supplementary file 7 — Source data Fig. 4 [file 44318_2025_505_MOESM7_ESM.zip › Figure 4/4F-G/control -Q.tif]

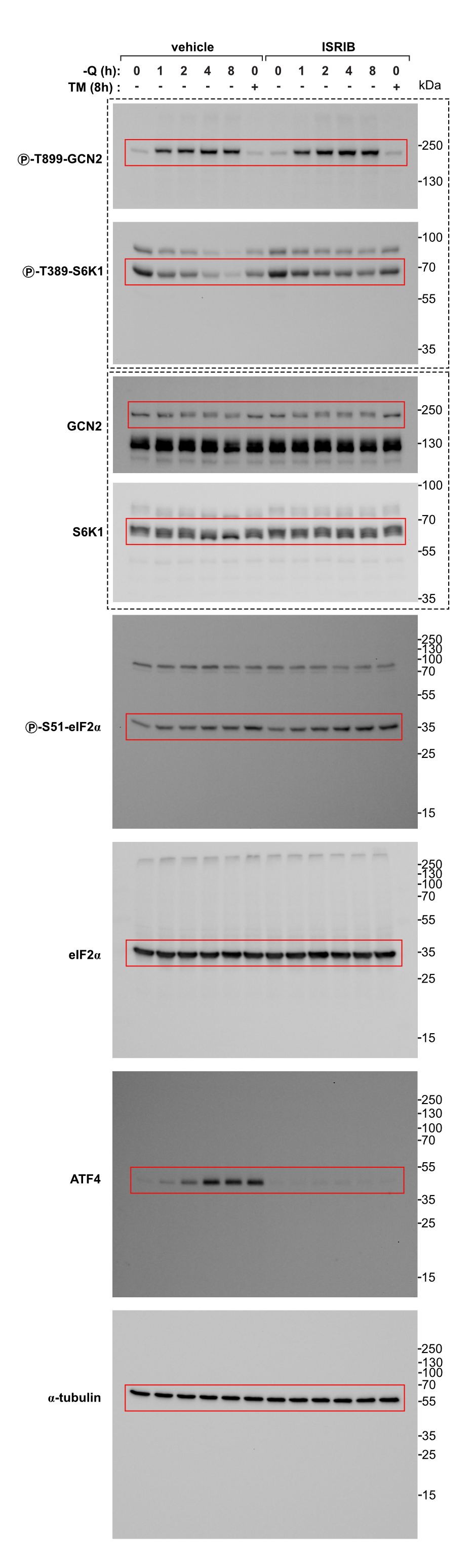

Supplement: Supplementary file 7 — Source data Fig. 4 [file 44318_2025_505_MOESM7_ESM.zip › Figure 4/4C-D/uncropped blots.tiff]

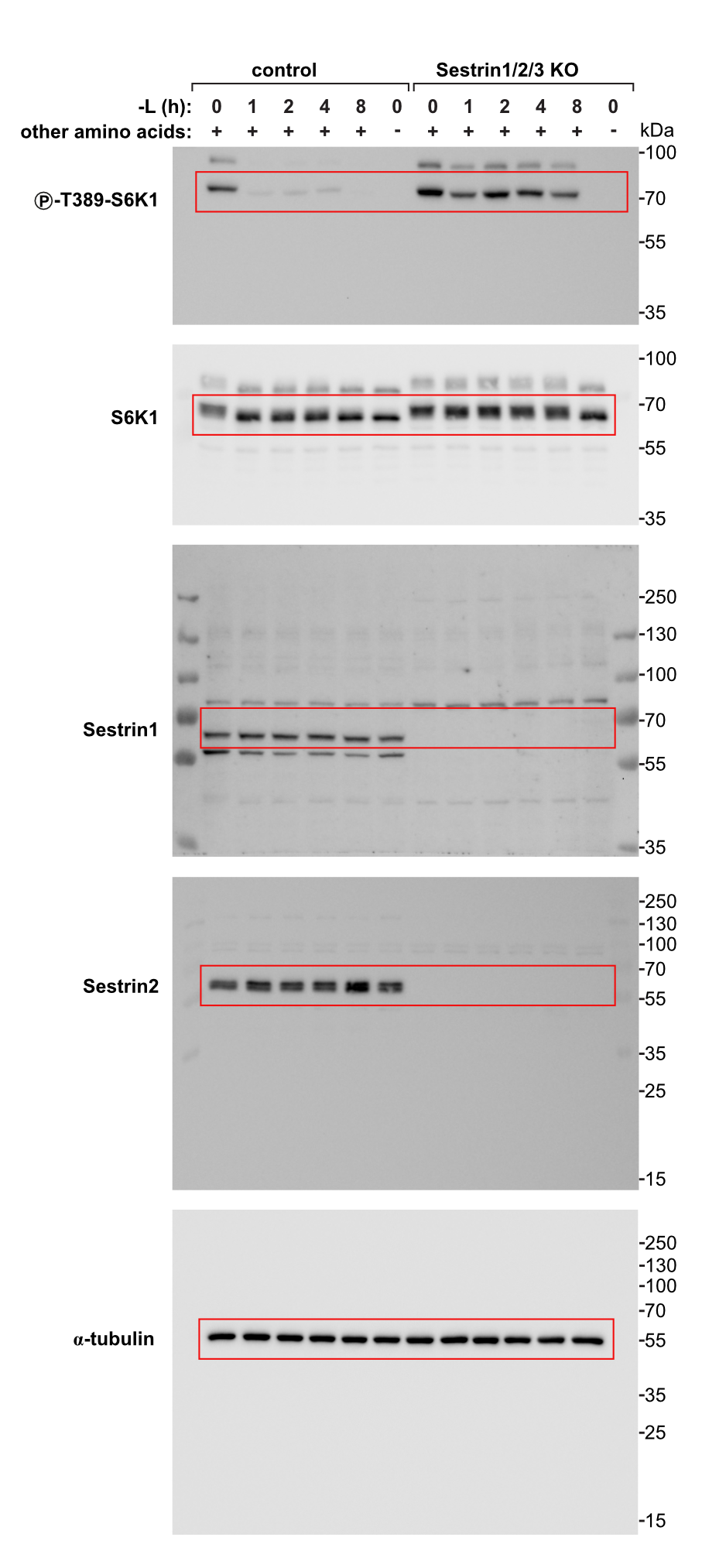

Supplement: Supplementary file 8 — Source data Fig. 5 [file 44318_2025_505_MOESM8_ESM.zip › Figure 5/5G-H/uncropped blots.tiff]

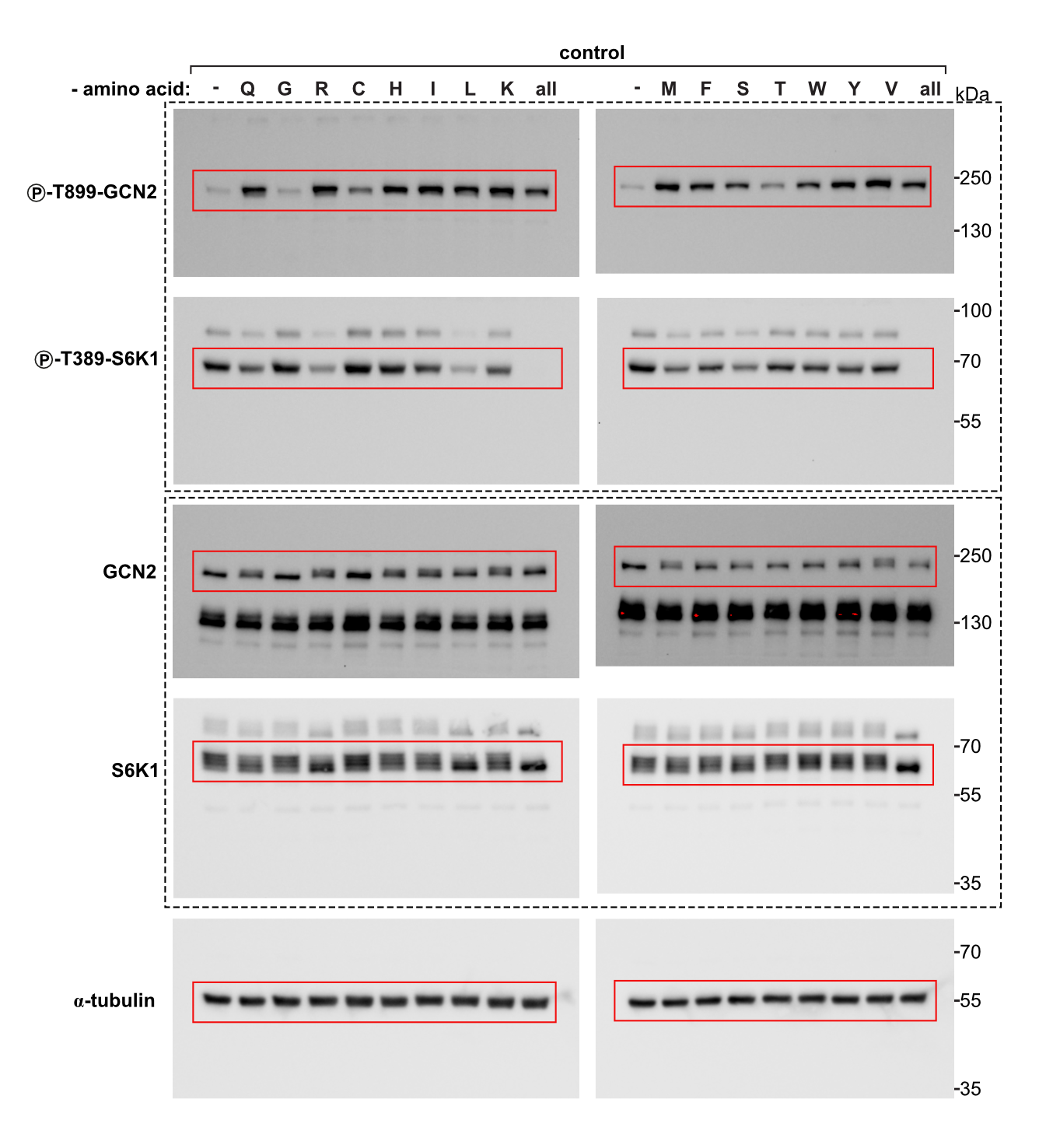

Supplement: Supplementary file 8 — Source data Fig. 5 [file 44318_2025_505_MOESM8_ESM.zip › Figure 5/5A-B/uncropped blots.tiff]

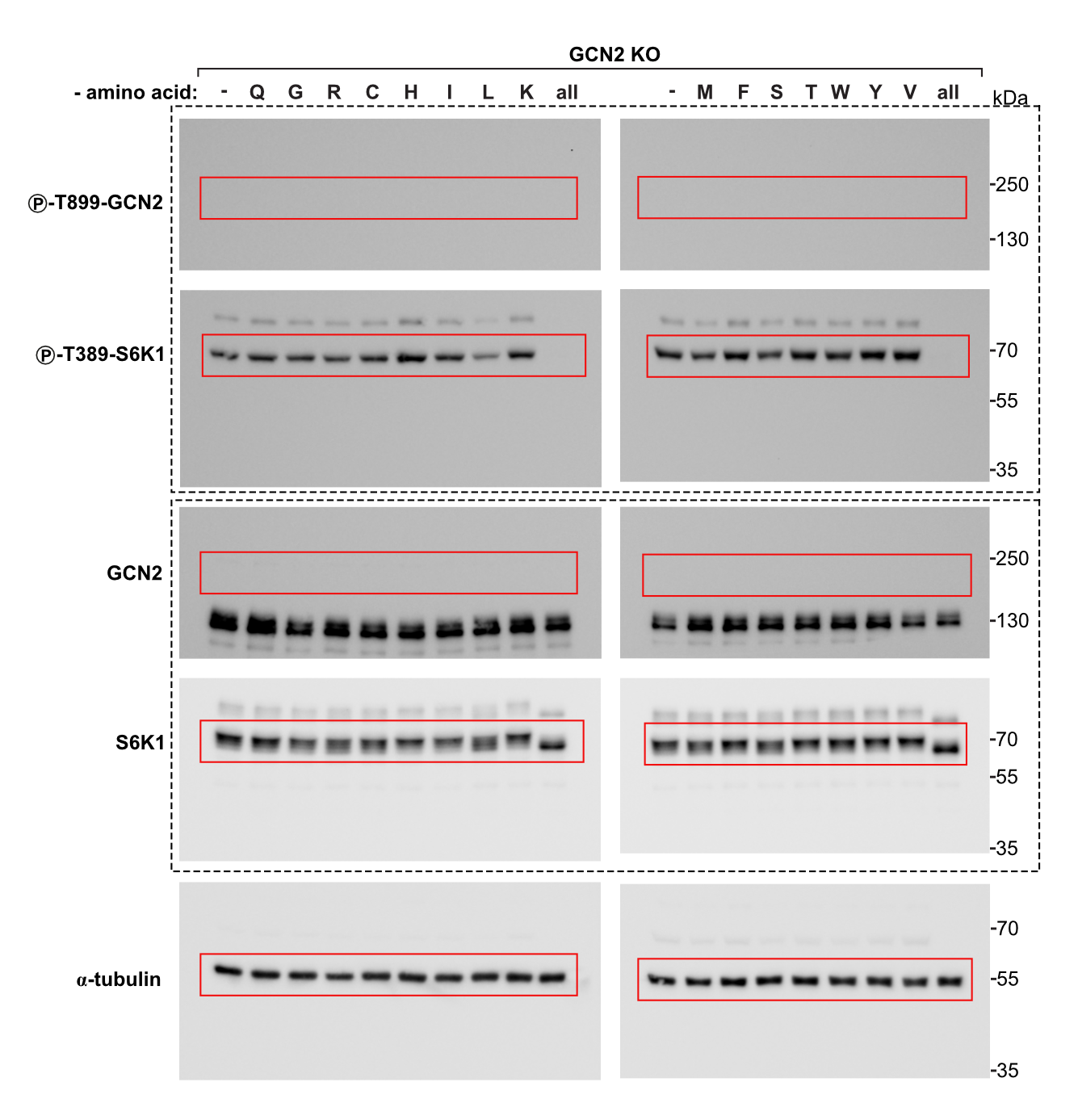

Supplement: Supplementary file 8 — Source data Fig. 5 [file 44318_2025_505_MOESM8_ESM.zip › Figure 5/5C-D/uncropped blots.tiff]

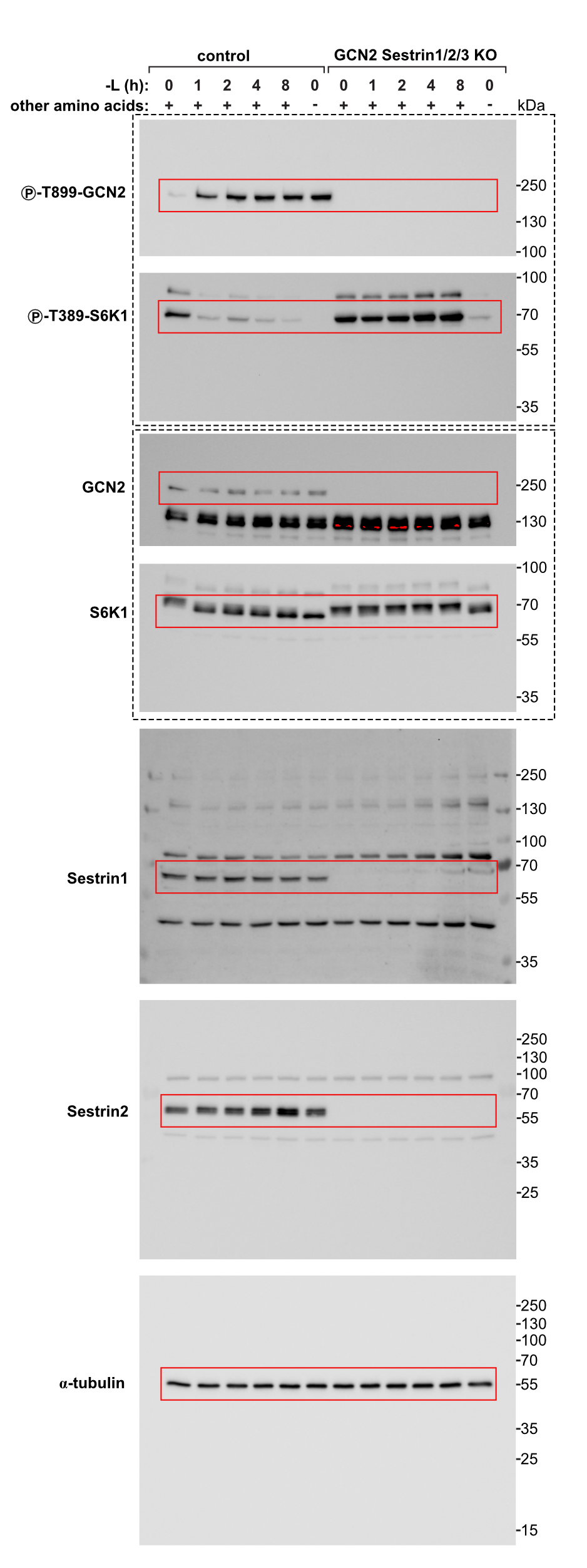

Supplement: Supplementary file 8 — Source data Fig. 5 [file 44318_2025_505_MOESM8_ESM.zip › Figure 5/5I-J/uncropped blots.tiff]

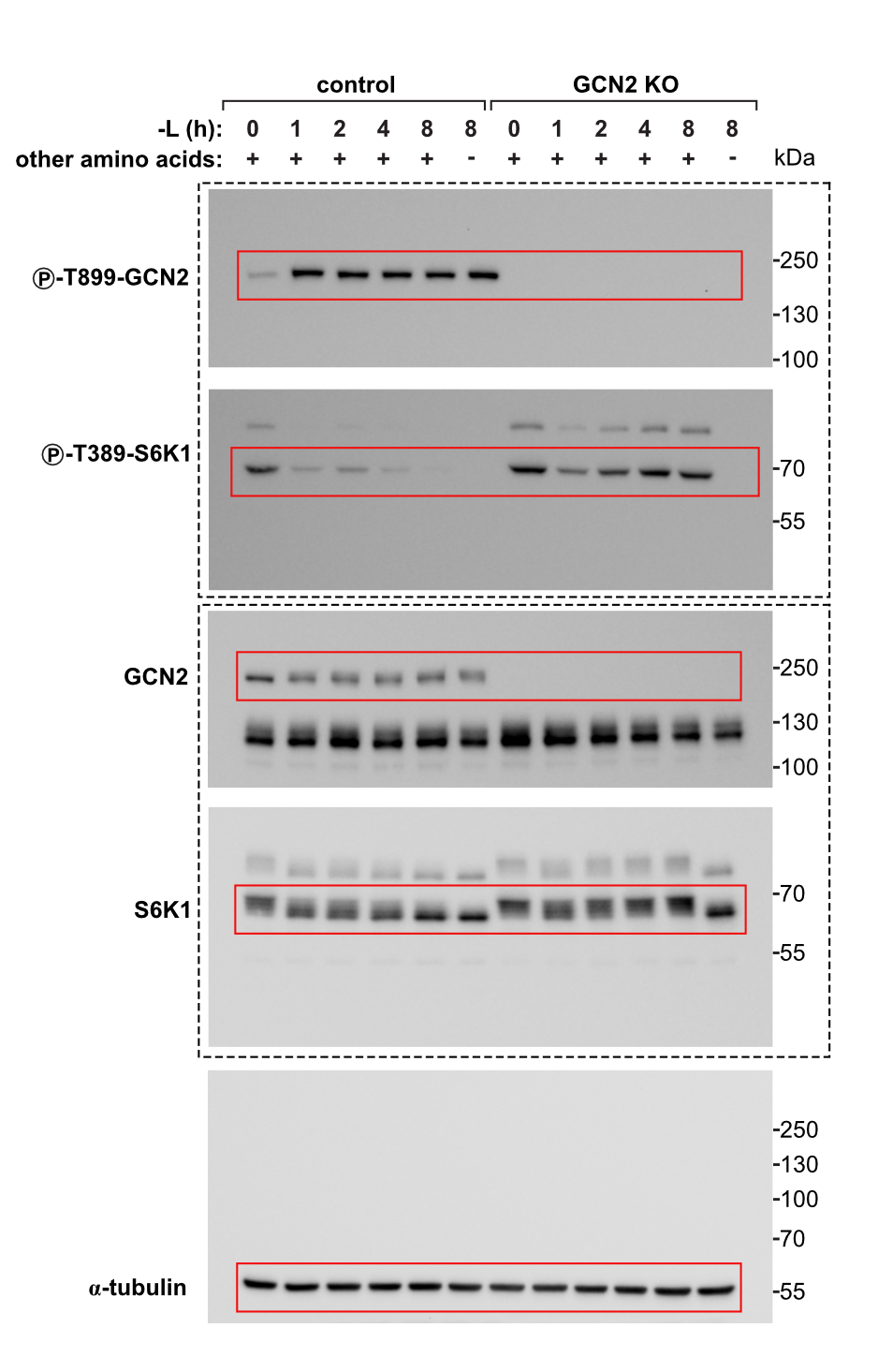

Supplement: Supplementary file 8 — Source data Fig. 5 [file 44318_2025_505_MOESM8_ESM.zip › Figure 5/5E-F/uncropped blots.tiff]

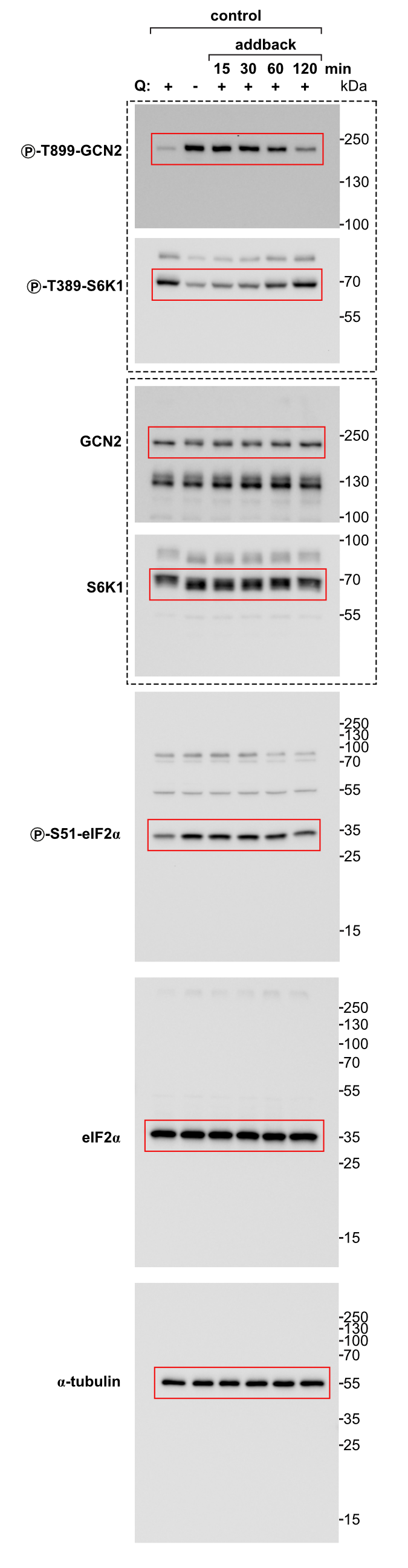

Supplement: Supplementary file 9 — Source data Fig. 6 [file 44318_2025_505_MOESM9_ESM.zip › Figure 6/6C-D/uncropped blots.tiff]

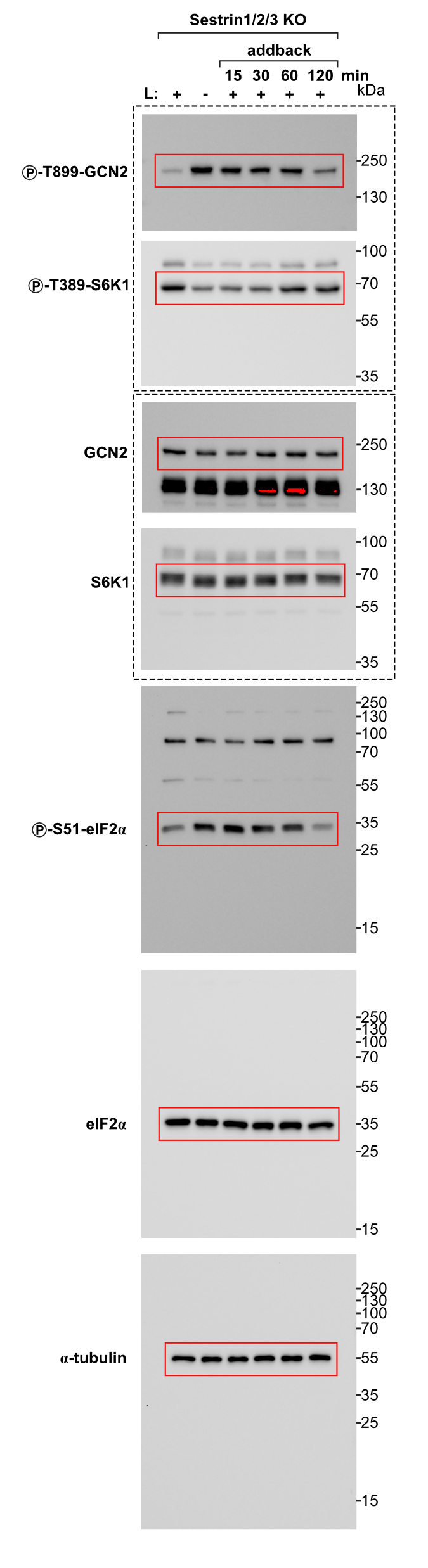

Supplement: Supplementary file 9 — Source data Fig. 6 [file 44318_2025_505_MOESM9_ESM.zip › Figure 6/6E-F/uncropped blots.tiff]

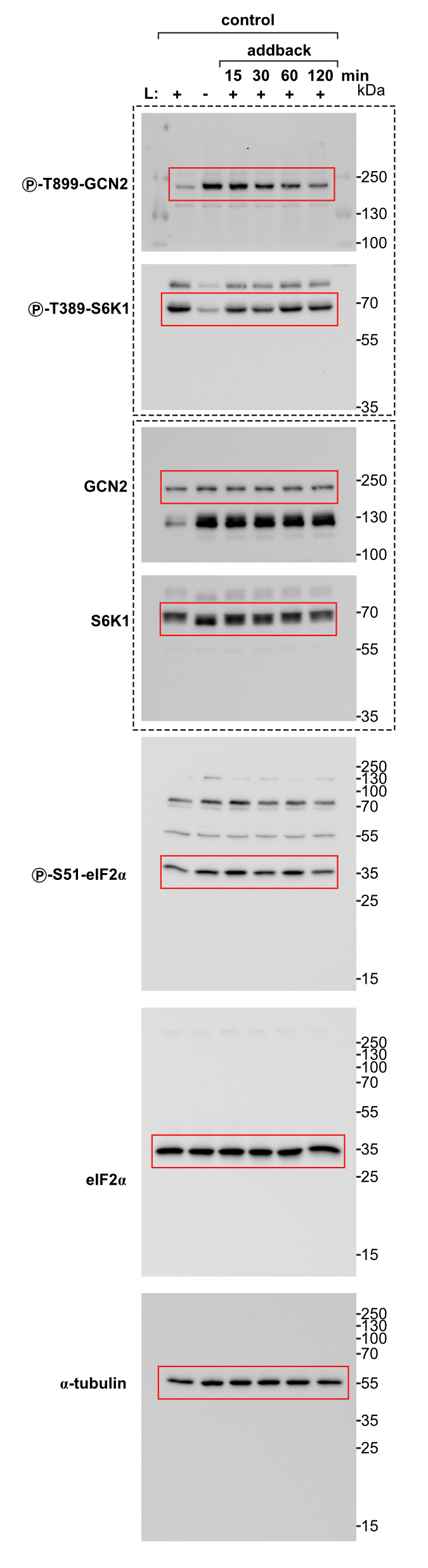

Supplement: Supplementary file 9 — Source data Fig. 6 [file 44318_2025_505_MOESM9_ESM.zip › Figure 6/6A-B/uncropped blots.tiff]
