# Supplementary material for: mTORC1 senses glutamine and other amino acids through GCN2
Source: EMBO J. 2025 Jul 21;44(17):4825–66. doi: 10.1038/s44318-025-00505-1 (PMC12402317; doi:10.1038/s44318-025-00505-1)
Supplement: Supplementary file 10 — Expanded View Figures [file 44318_2025_505_MOESM10_ESM.pdf]

Expanded View Figures

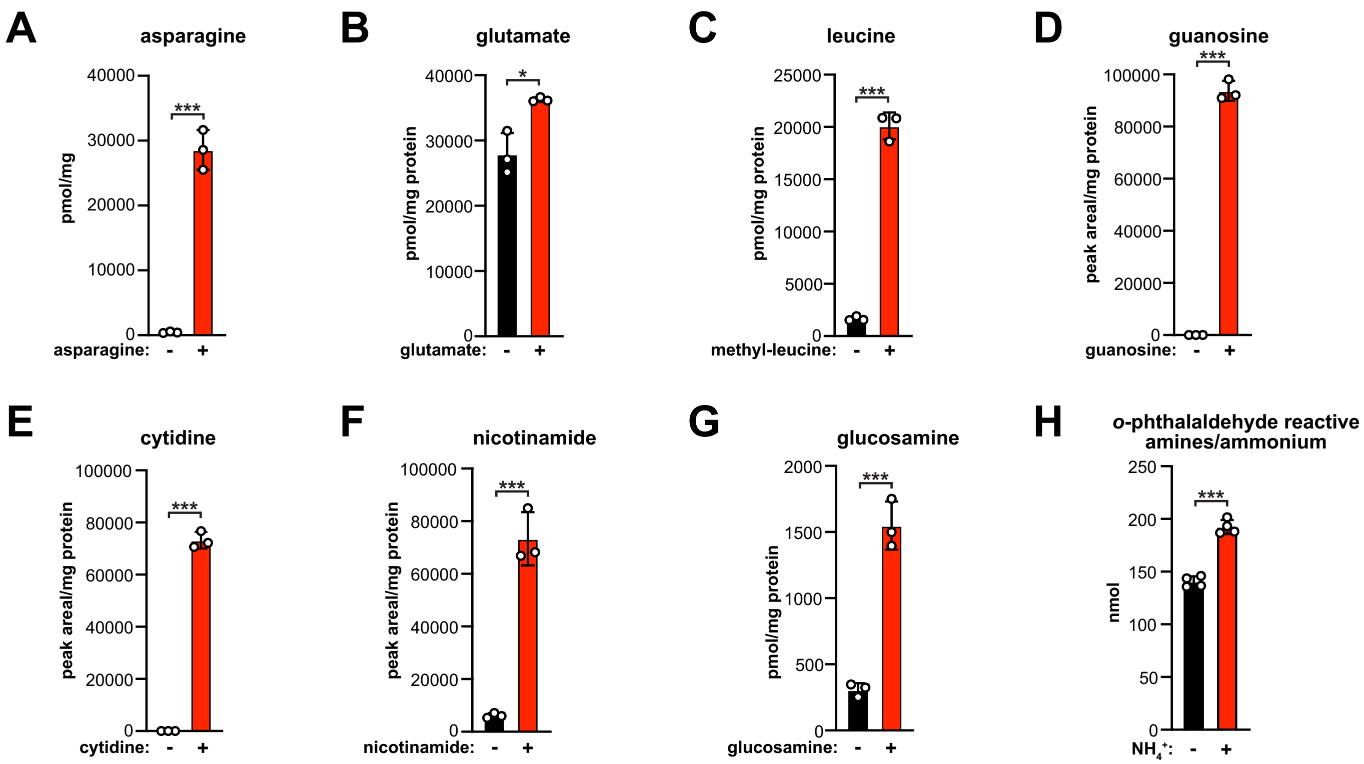

Figure EV1. Measurement of intracellular downstream glutamine metabolites and metabolite precursors upon exogenous addition.

(**A–H**) Exogenous addition of the metabolites in Fig. 1B results in a corresponding increase in their intracellular levels in control HEK293T cells. In the case of methyl-leucine, intracellular levels of non-esterified leucine were determined. Ammonium levels in (**H**) were measured as *o*-phthalaldehyde-reactivity. All metabolites were added for 1 h at 4 mM to cells cultured in regular DMEM. Data are expressed as either pmol amino acid per mg protein (**A–C**, **G**), peak area normalized per mg protein (**D–F**) or nmol per sample (**H**). Bar height: average, error bars: standard deviation, $n = 3$ (**A–G**) or 4 (**H**) biological replicates. Two-tailed unpaired *t* test. $^*P < 0.05$, $^{***}P < 0.001$.

© The Author(s)

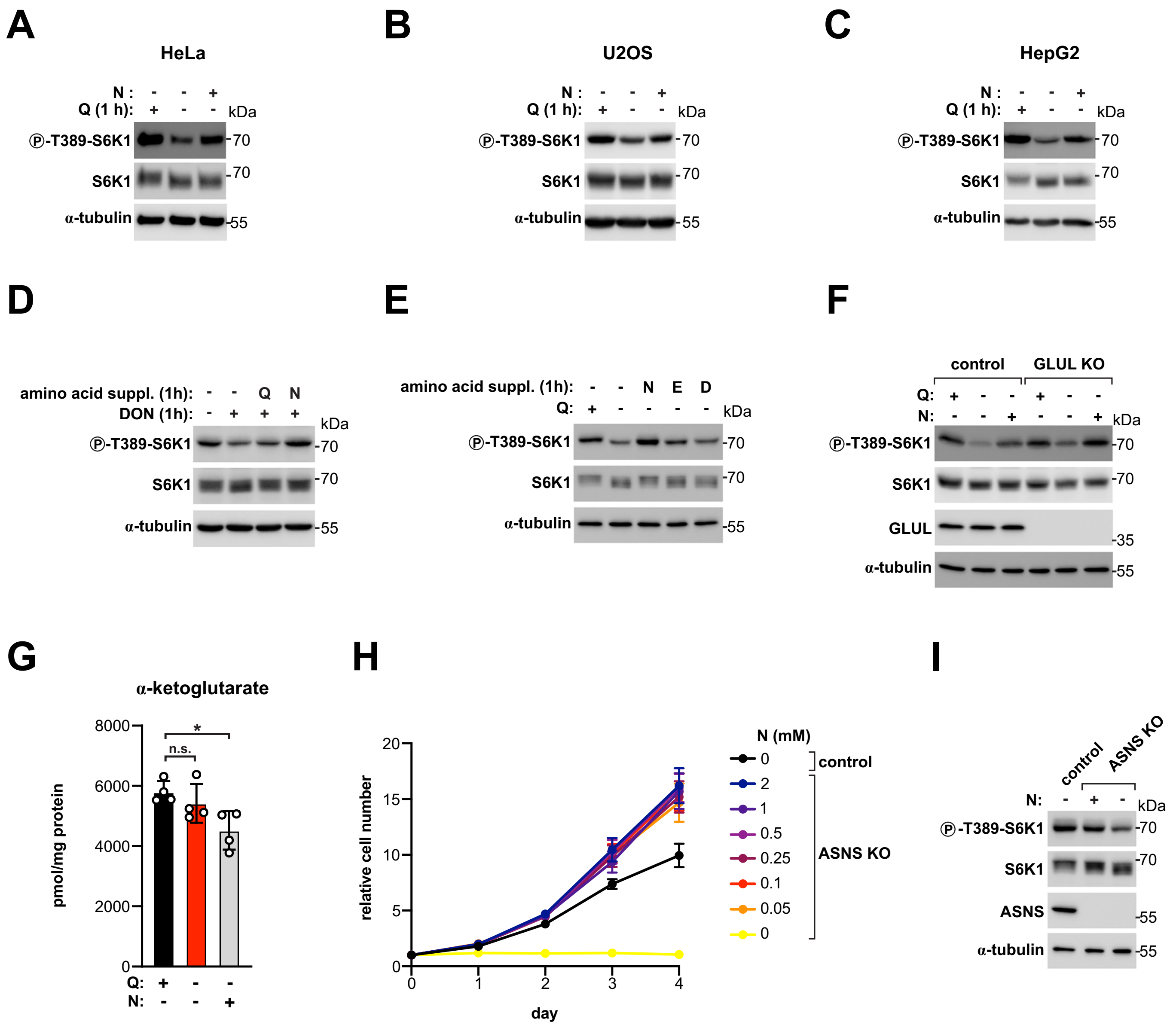
A
HeLa
N :
Q (1 h):
kDa
Ⓟ-T389-S6K1
S6K1
α-tubulin
B
U2OS
C
HepG2
D
amino acid suppl. (1h):
DON (1h):
E
F
control
GLUL KO
GLUL
G
α-ketoglutarate
pmol/mg protein
n.s.
*
H
relative cell number
day
N (mM)
control
ASNS KO
I
ASNS

Figure EV2. Asparagine is both necessary and sufficient to maintain mTORC1 activity downstream of glutamine metabolism.

(**A–C**) Asparagine rescues mTORC1 inhibition upon acute glutamine depletion in multiple cell lines. Immunoblot from HeLa (**A**), U2OS (**B**) and HepG2 (**C**) cells incubated for 1 h in regular DMEM, glutamine-free DMEM or glutamine-free DMEM supplemented with 250 μM asparagine. Q: glutamine, N: asparagine. Representative of 5 biological replicates. The same samples were loaded on different gels and blotted in parallel with the indicated antibodies. Protein concentration was controlled by blotting α-tubulin on a separate gel. (**D**) Supplementation with asparagine rescues mTORC1 activity upon inhibition of glutamine metabolism with DON. Immunoblot from HEK293T cells treated for 1 h with DON (100 μM) and incubated in regular DMEM or DMEM supplemented with additional glutamine (Q, 4 mM) or with asparagine (N, 4 mM). Representative of 2 biological replicates. The same samples were loaded on different gels and blotted in parallel with the indicated antibodies. Protein concentration was controlled by blotting α-tubulin on a separate gel. (**E**) Neither glutamate nor aspartate rescue mTORC1 inhibition upon glutamine removal. Immunoblot from HEK293T cells incubated for 1 h in regular DMEM, glutamine-free DMEM or glutamine-free DMEM supplemented with 4 mM asparagine (N), glutamate (E) or aspartate (D). Representative of 3 biological replicates. The same samples were loaded on different gels and blotted in parallel with the indicated antibodies. Protein concentration was controlled by blotting α-tubulin on a separate gel. (**F**) Asparagine supplementation rescues mTORC1 inhibition in glutamine synthetase (GLUL)-knockout cells starved of glutamine. Immunoblot from control and GLUL KO HEK293T cells incubated for 1 h in regular DMEM, glutamine-free DMEM or glutamine-free DMEM supplemented with 250 μM asparagine. Q: glutamine, N: asparagine. Representative of 3 biological replicates. The same samples were loaded on different gels and blotted in parallel with the indicated antibodies. Protein concentration was controlled by blotting α-tubulin on a separate gel. (**G**) Neither glutamine depletion nor asparagine supplementation affect α-ketoglutarate levels. α-ketoglutarate was measured in control HEK293T cells incubated for 1 h in regular DMEM, glutamine-free DMEM or glutamine-free DMEM supplemented with 250 μM asparagine. Q: glutamine, N: asparagine. Data are expressed as pmol α-ketoglutarate per mg protein. Bar height: average, error bars: standard deviation, $n = 4$ biological replicates. One-way ANOVA and Tukey's post hoc test. *$p < 0.05$, n.s.: non significant. (**H**) ASNS KO cells require asparagine supplementation to proliferate. Relative cell numbers for control HEK293T or ASNS KO cells cultured over four days in regular DMEM (which does not contain asparagine) or in DMEM supplemented with the indicated asparagine (N) concentrations. Cell numbers on the day of seeding (day 0) are set to 1. Circle: average, error bars: standard deviation, $n = 5$ technical replicates. (**I**) Removal of asparagine inhibits mTORC1. Immunoblot from control and ASNS KO HEK293T cells incubated for 1 h in DMEM supplemented with 250 μM asparagine (N) or in regular DMEM (which does not contain asparagine). Representative of 3 biological replicates. The same samples were loaded on different gels and blotted in parallel with the indicated antibodies. Protein concentration was controlled by blotting α-tubulin on a separate gel.

© The Author(s)

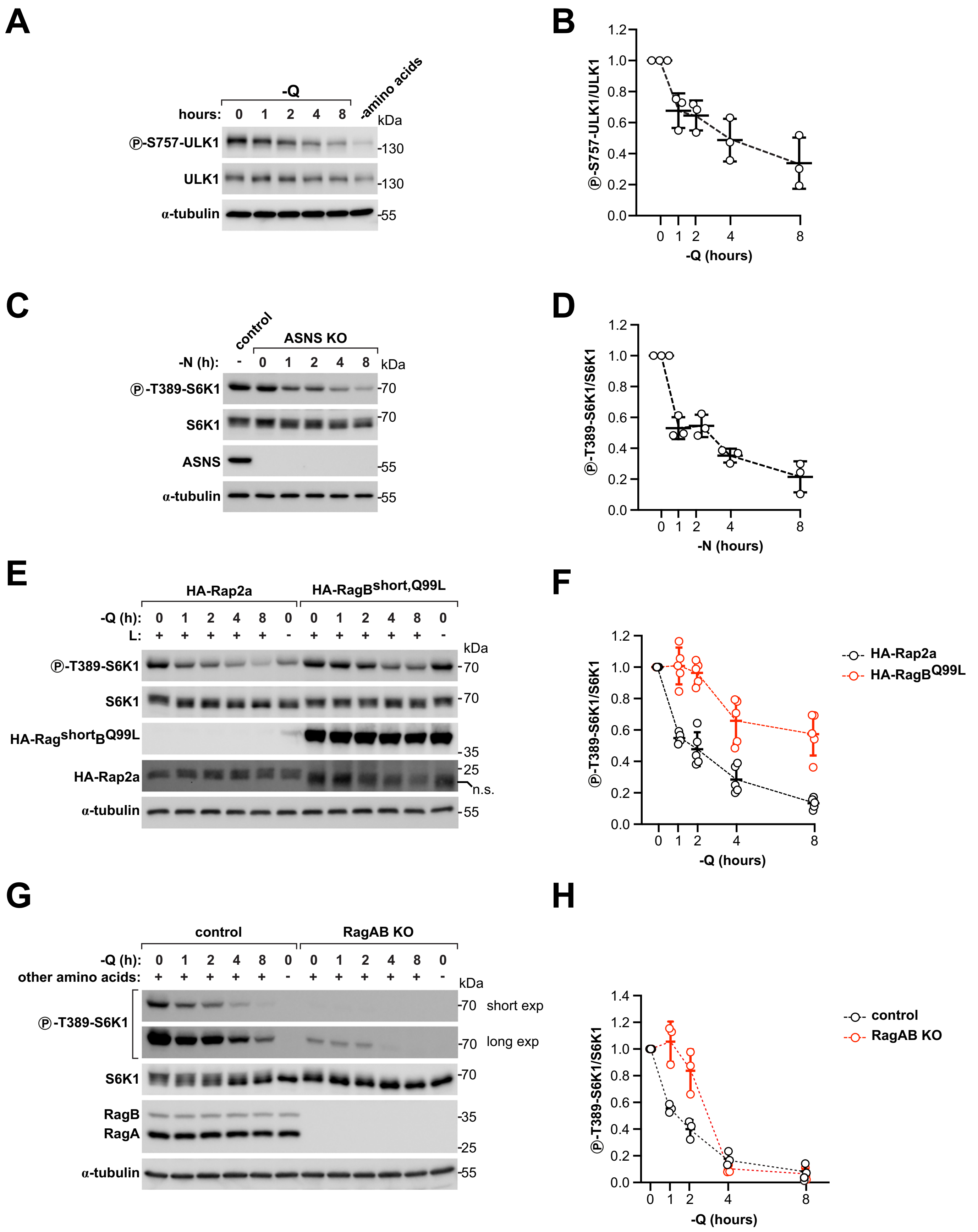
A
-Q
hours: 0 1 2 4 8 -amino acids
kDa
Ⓟ-S757-ULK1 -130
ULK1 -130
α-tubulin -55
B
Ⓟ-S757-ULK1/ULK1
-Q (hours)
C
control
ASNS KO
-N (h): - 0 1 2 4 8 kDa
Ⓟ-T389-S6K1 -70
S6K1 -70
ASNS -55
α-tubulin -55
D
Ⓟ-T389-S6K1/S6K1
-N (hours)
E
HA-Rap2a
HA-RagBshort,Q99L
-Q (h): 0 1 2 4 8 0 0 1 2 4 8 0
L: + + + + + - + + + + + -
kDa
Ⓟ-T389-S6K1 -70
S6K1 -70
HA-RagshortBQ99L -35
HA-Rap2a -25 n.s.
α-tubulin -55
F
HA-Rap2a
HA-RagBQ99L
Ⓟ-T389-S6K1/S6K1
-Q (hours)
G
control
RagAB KO
-Q (h): 0 1 2 4 8 0 0 1 2 4 8 0
other amino acids: + + + + + - + + + + + -
kDa
Ⓟ-T389-S6K1 -70 short exp -70 long exp
S6K1 -70
RagB -35
RagA -25
α-tubulin -55
H
control
RagAB KO
Ⓟ-T389-S6K1/S6K1
-Q (hours)

 © The Author(s)

Figure EV3. Glutamine is sensed through both Rag-dependent and Rag-independent mechanisms.

(**A**, **B**) Glutamine removal causes dephosphorylation of the mTORC1 substrate ULK1. Immunoblot from control HEK293T cells incubated in regular or glutamine-free DMEM for the indicated time points. As a control, cells were incubated in amino acid-free DMEM for 8 h. Representative example (**A**) of 3 biological replicates quantified in (**B**). The same samples were loaded on different gels and blotted in parallel with the indicated antibodies. Protein concentration was controlled by blotting α-tubulin on a separate gel. Glutamine-rich condition is set to 1. Q: glutamine, line: average, error bars: standard deviation. (**C**, **D**) Asparagine removal inactivates mTORC1 progressively over time. Immunoblot from control and ASNS KO HEK293T cells incubated in DMEM supplemented with 250 μM asparagine (N) or in regular DMEM (which does not contain asparagine) for the indicated time points. Representative example (**C**) of 3 biological replicates quantified in (**D**). The same samples were loaded on different gels and blotted in parallel with the indicated antibodies. Protein concentration was controlled by blotting α-tubulin on a separate gel. Asparagine-rich condition is set to 1. N: asparagine, line: average, error bars: standard deviation. (**E**, **F**) Expression of constitutively active $RagB^{short}$ prevents acute (1–2 h) but not late mTORC1 inhibition after glutamine removal. Immunoblot from HEK293T stably transfected with a control protein (HA-Rap2a) or with HA-tagged constitutively active $RagB^{short}$ (Q99L) and incubated in regular or glutamine-free DMEM for the indicated time points. As a control, cells were incubated in leucine-free DMEM for 2 h. Q: glutamine, L: leucine. n.s.: non-specific band. Representative example (**C**) of 5 biological replicates quantified in (**D**). The same samples were loaded on different gels and blotted in parallel with the indicated antibodies. Protein concentration was controlled by blotting α-tubulin on a separate gel. Glutamine-rich condition for each genotype is set to 1. Q: glutamine, line: average, error bars: standard deviation. (**G**, **H**) Double deletion of RagA and RagB prevents acute (1–2 h) but not late mTORC1 inhibition after glutamine removal. Immunoblot from control and RagAB-knockout HEK293T cells incubated in regular or glutamine-free DMEM for the indicated time points. As a control, cells were incubated in amino acid-free DMEM for 8 h. Representative example (**G**) of 3 biological replicates quantified in (**H**). The same samples were loaded on different gels and blotted in parallel with the indicated antibodies. Protein concentration was controlled by blotting α-tubulin on a separate gel. Glutamine-rich condition for each genotype is set to 1. Q: glutamine, exp: exposure, line: average, error bars: standard deviation.

© The Author(s)

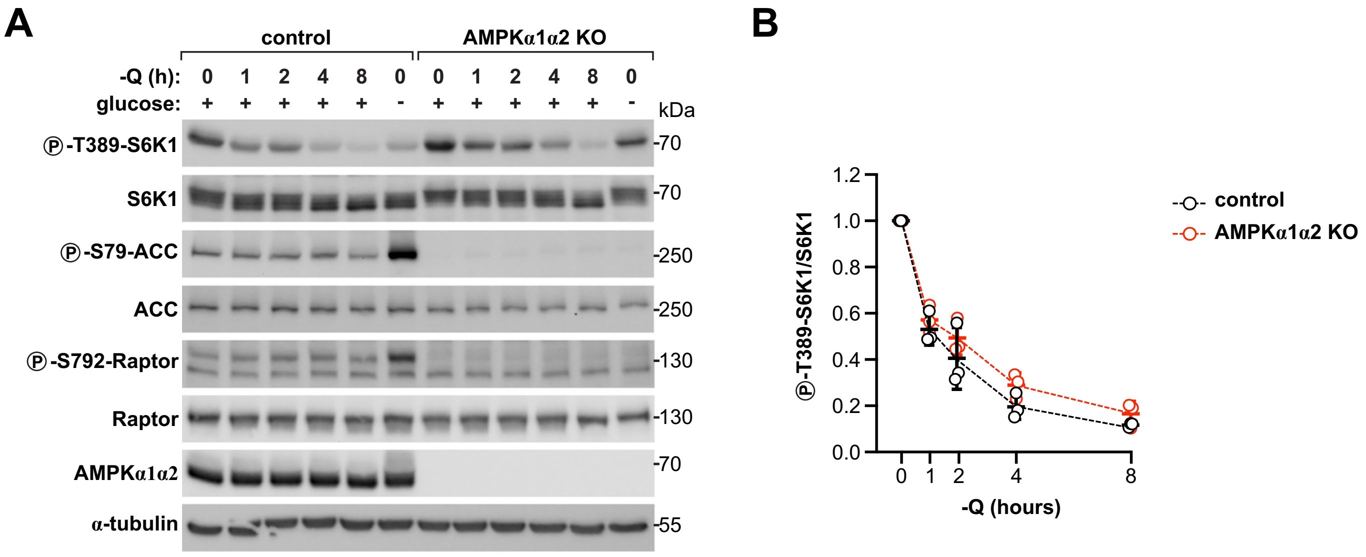

Figure EV4. Glutamine is not sensed through AMPK.

(**A**, **B**) Inactivation of mTORC1 upon glutamine removal is independent of AMPK. Immunoblot from control or AMPKα1/α2-knockout HEK293T cells incubated in regular or glutamine-free DMEM for the indicated time points. As a control, cells were incubated in glucose-free DMEM for 2 h. Q: glutamine. Representative example (**A**) of 3 biological replicates quantified in (**B**). The same samples were loaded on different gels and blotted in parallel with the indicated antibodies. Protein concentration was controlled by blotting α-tubulin on a separate gel. Glutamine-rich condition for each genotype is set to 1. Line: average, error bars: standard deviation.

 © The Author(s)

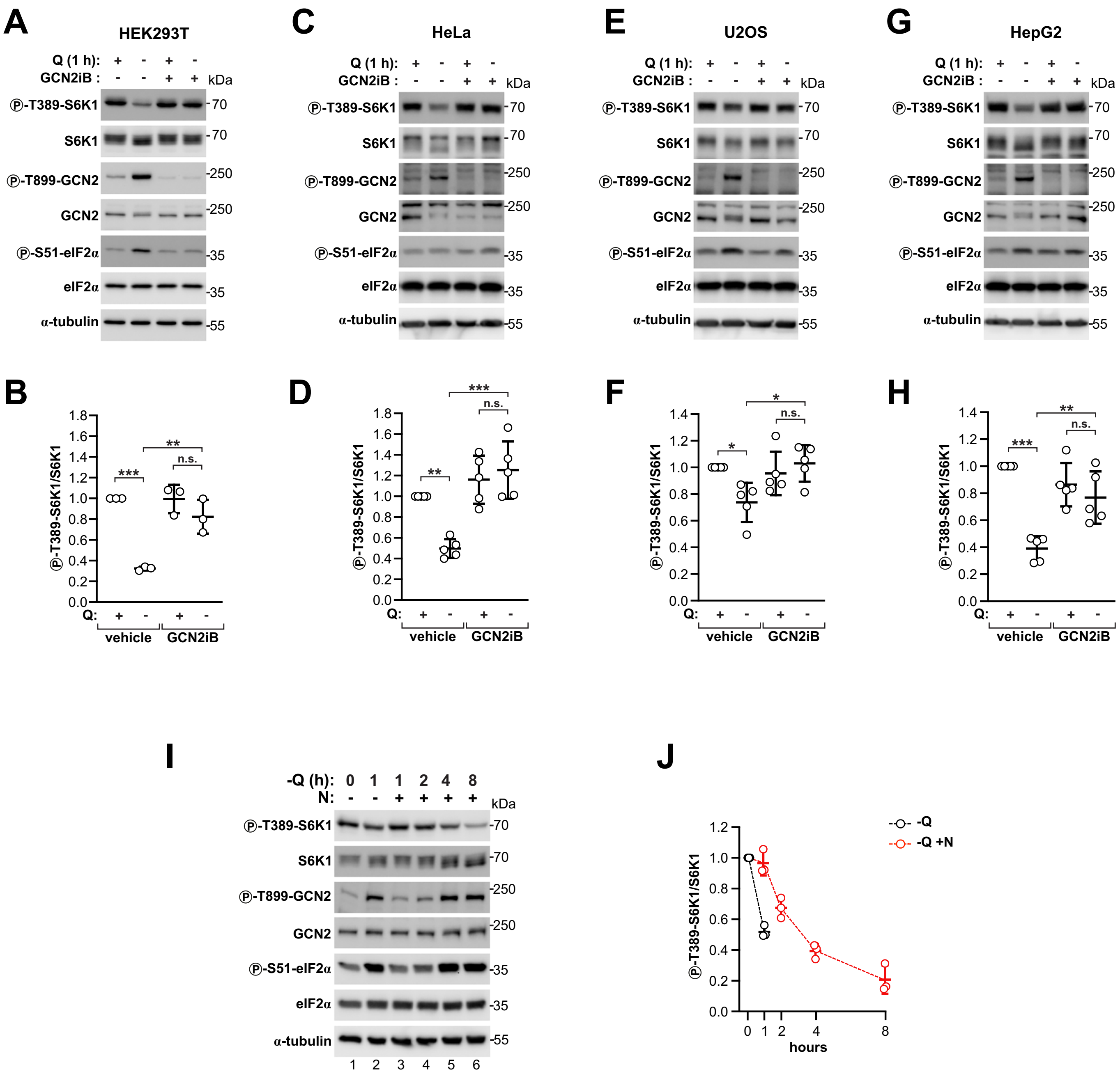

Figure EV5. Glutamine is sensed through GCN2 in multiple cell lines.

(**A–H**) mTORC1 inhibition upon acute glutamine depletion for 1 h requires GCN2. Immunoblot from HEK293T (**A**, **B**), HeLa (**C**, **D**), U2OS (**E**, **F**) and HepG2 (**G**, **H**) cells treated with vehicle (DMSO) or GCN2iB (1 μM) and incubated in regular or glutamine-free DMEM for 1 h. Representative example of 3 (**A**) or 5 (**C**, **E**, **G**) biological replicates quantified in (**B**, **D**, **F**, **H**). The same samples were loaded on different gels and blotted in parallel with the indicated antibodies. Protein concentration was controlled by blotting α-tubulin on a separate gel. Vehicle-treated unstarved cells are set to 1. Q: glutamine, line: average, error bars: standard deviation. One-way ANOVA and Tukey's post hoc test. *$P < 0.05$, **$P < 0.01$, ***$P < 0.001$, n.s.: non significant. (**I**, **J**) Asparagine supplementation rescues GCN2 activation and mTORC1 inhibition acutely, but not during prolonged glutamine depletion. Immunoblot from control HEK293T cells incubated in regular or glutamine-free DMEM for the indicated time points and supplemented with 250 μM asparagine. Representative example (**I**) of 3 biological replicates quantified in (**J**). The same samples were loaded on different gels and blotted in parallel with the indicated antibodies. Protein concentration was controlled by blotting α-tubulin on a separate gel. Glutamine-rich condition is set to 1 and relative phosphorylation of S6K1 upon glutamine starvation for 1 h or glutamine starvation and supplementation of asparagine from one to eight hours is shown. Q: glutamine, N: asparagine, line: average, error bars: standard deviation.

© The Author(s)

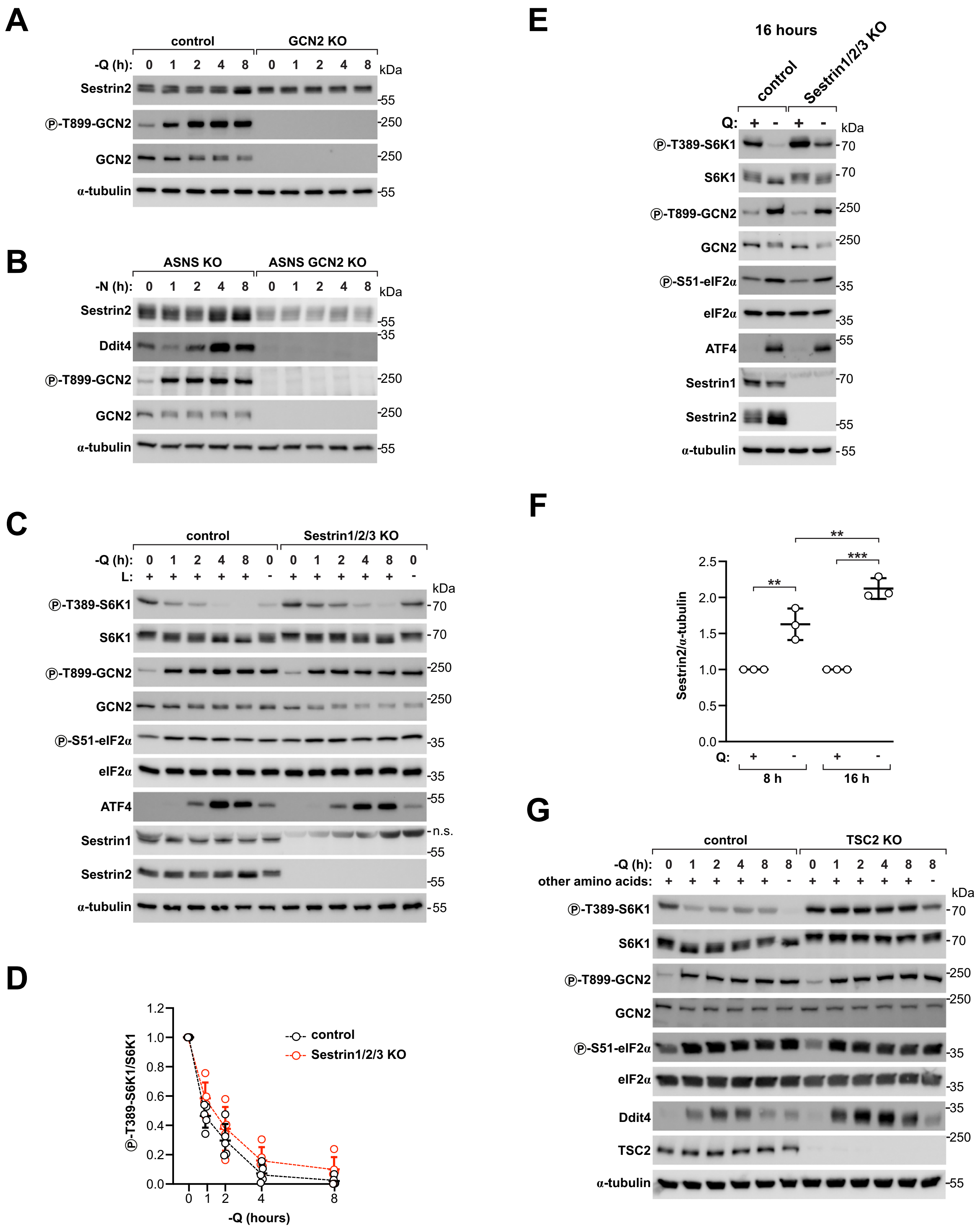
A
control
GCN2 KO
-Q (h): 0 1 2 4 8 0 1 2 4 8
kDa
Sestrin2
Ⓟ-T899-GCN2
GCN2
α-tubulin
55
250
250
55
B
ASNS KO
ASNS GCN2 KO
-N (h): 0 1 2 4 8 0 1 2 4 8
kDa
Sestrin2
Ddit4
Ⓟ-T899-GCN2
GCN2
α-tubulin
C
control
Sestrin1/2/3 KO
-Q (h): 0 1 2 4 8 0 0 1 2 4 8 0
L: + + + + + - + + + + + -
kDa
Ⓟ-T389-S6K1
S6K1
Ⓟ-T899-GCN2
GCN2
Ⓟ-S51-eIF2α
eIF2α
ATF4
Sestrin1
Sestrin2
α-tubulin
n.s.
D
Ⓟ-T389-S6K1/S6K1
-Q (hours)
control
Sestrin1/2/3 KO
E
16 hours
control
Sestrin1/2/3 KO
Q: + - + -
kDa
Ⓟ-T389-S6K1
S6K1
Ⓟ-T899-GCN2
GCN2
Ⓟ-S51-eIF2α
eIF2α
ATF4
Sestrin1
Sestrin2
α-tubulin
F
Sestrin2/α-tubulin
Q: + - + -
8 h
16 h
**
***
G
control
TSC2 KO
-Q (h): 0 1 2 4 8 8 0 1 2 4 8 8
other amino acids: + + + + + - + + + + + -
kDa
Ⓟ-T389-S6K1
S6K1
Ⓟ-T899-GCN2
GCN2
Ⓟ-S51-eIF2α
eIF2α
Ddit4
TSC2
α-tubulin

 © The Author(s)

Figure EV6. Induction of Sestrin2 downstream of the ISR is involved in mTORC1 inhibition during overnight glutamine deprivation.

(**A**) Sestrin2 protein levels increase at late time points after glutamine deprivation in a GCN2-dependent manner. Immunoblot from control or GCN2 KO HEK293T cells incubated in regular or glutamine-free DMEM for the indicated time points. Q: glutamine. Representative of 3 biological replicates. The same samples were loaded on different gels and blotted in parallel with the indicated antibodies. Protein concentration was controlled by blotting α-tubulin on a separate gel. (**B**) Sestrin2 and Ddit4 protein levels increase upon asparagine deprivation in a GCN2-dependent manner. Immunoblot from ASNS KO or ASNS GCN2 KO HEK293T cells incubated in DMEM supplemented with 250 μM asparagine (N) or in regular DMEM (which does not contain asparagine) for the indicated time points. Representative of 3 biological replicates. The same samples were loaded on different gels and blotted in parallel with the indicated antibodies. Protein concentration was controlled by blotting α-tubulin on a separate gel. (**C**, **D**) Sestrin1/2/3 do not significantly contribute to the inhibition of mTORC1 within the first 8 h of glutamine removal. Immunoblot from control and Sestrin1/2/3 KO HEK293T cells incubated in regular or glutamine-free DMEM for the indicated time points. As a control, cells were incubated in leucine-free DMEM for 2 h. Deletion of Sestrin3 was determined through sequencing of the corresponding genomic locus, given the poor quality of the Sestrin3 antibodies tested. Q: glutamine, L: leucine. n.s.: non-specific band. Representative experiment (**B**) of 5 biological replicates quantified in (**C**). The same samples were loaded on different gels and blotted in parallel with the indicated antibodies. Protein concentration was controlled by blotting α-tubulin on a separate gel. Glutamine-rich condition is set to 1 for all genotypes. Line: average, error bars: standard deviation. (**E**, **F**) Sestrin1/2/3 contribute towards maintaining mTORC1 repression upon prolonged removal of glutamine. Immunoblot from control HEK293T and Sestrin1/2/3 KO cells incubated in regular or glutamine-free DMEM overnight (16 h) (**D**) and quantification of Sestrin2 induction at 16 h and at 8 h in the experiment in (**A**). The same samples were loaded on different gels and blotted in parallel with the indicated antibodies. Protein concentration was controlled by blotting α-tubulin on a separate gel. Q: glutamine. Representative of 3 biological replicates. Glutamine-rich condition for each time point is set to 1. Circle: average, error bars: standard deviation. One-way ANOVA and Tukey's post hoc test. $^{**}P < 0.01$, $^{***}P < 0.001$. (**G**) mTORC1 inhibition upon glutamine depletion requires the TSC complex. Immunoblot from control or TSC2 KO MEF cells incubated in regular or glutamine-free DMEM for the indicated time points. As a control, cells were treated with amino acid-free DMEM for 8 h. Q: glutamine. Representative example of 3 biological replicates. The same samples were loaded on different gels and blotted in parallel with the indicated antibodies. Protein concentration was controlled by blotting α-tubulin on a separate gel.

© The Author(s)

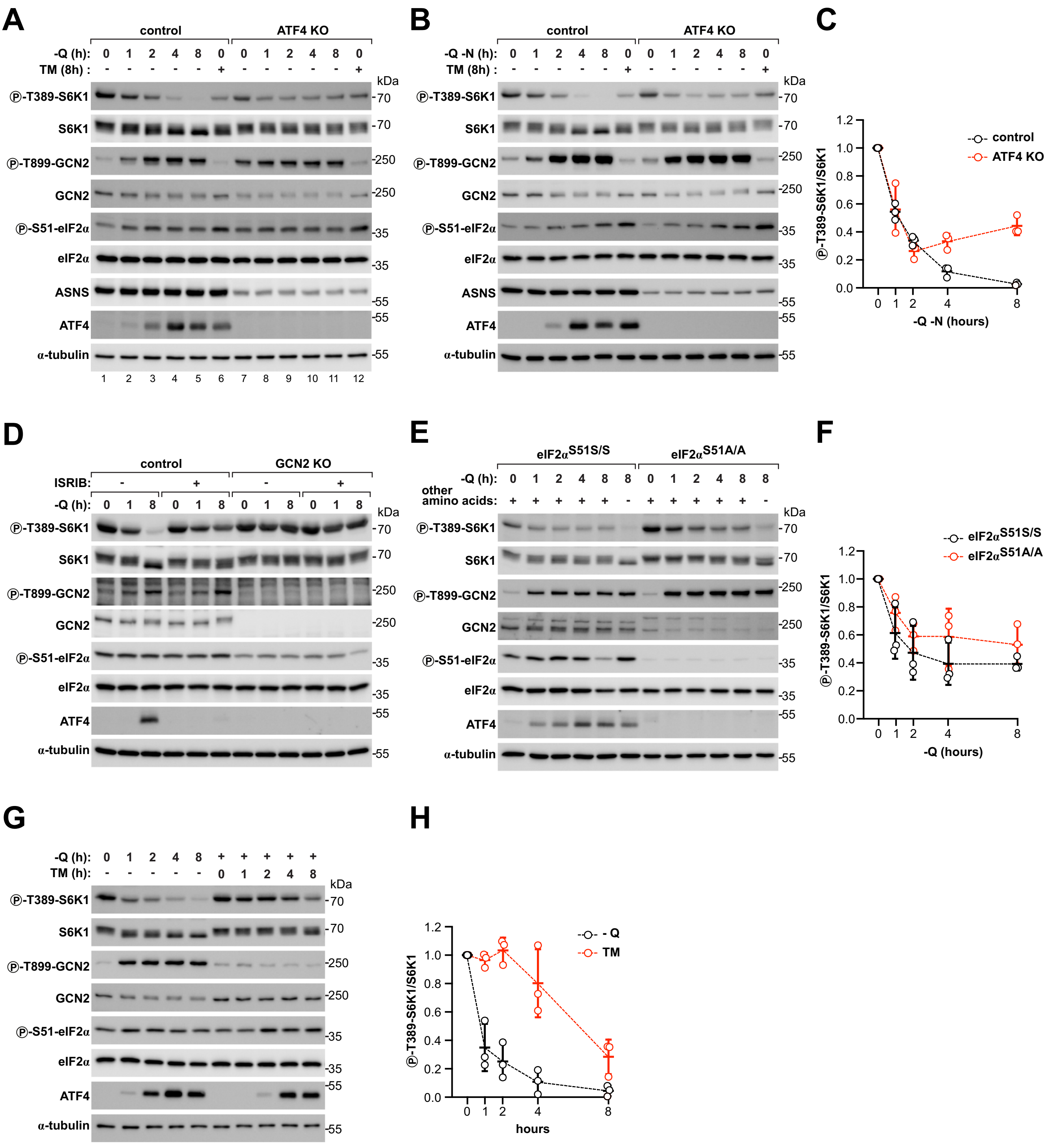
A
control
ATF4 KO
-Q (h): 0 1 2 4 8 0 0 1 2 4 8 0
TM (8h) : - - - - - + - - - - - +
kDa
Ⓟ-T389-S6K1
S6K1
Ⓟ-T899-GCN2
GCN2
Ⓟ-S51-eIF2α
eIF2α
ASNS
ATF4
α-tubulin
1 2 3 4 5 6 7 8 9 10 11 12
B
-Q -N (h):
TM (8h) :
C
Ⓟ-T389-S6K1/S6K1
control
ATF4 KO
-Q -N (hours)
D
control
GCN2 KO
ISRIB:
-Q (h):
E
eIF2α S51S/S
eIF2α S51A/A
other amino acids:
F
-Q (hours)
G
-Q (h):
TM (h):
H
- Q
TM
hours

Figure EV7. mTORC1 inhibition upon acute glutamine depletion does not require the ISR.

(**A**) Late but not acute mTORC1 inhibition upon glutamine deprivation requires ATF4. Immunoblot from control or ATF4 KO HEK293T cells incubated in regular (without asparagine) or glutamine-free DMEM for the indicated time points. As a control, cells were treated with tunicamycin (TM, 1 µg/ml) for 8 h. Q: glutamine. Representative of 3 biological replicates. The same samples were loaded on different gels and blotted in parallel with the indicated antibodies. Protein concentration was controlled by blotting α-tubulin on a separate gel. (**B**, **C**) Late but not acute mTORC1 inhibition upon combined glutamine and asparagine deprivation requires ATF4. Immunoblot from control or ATF4 KO HEK293T cells incubated in regular DMEM supplemented with 250 µM asparagine (N) or in glutamine-free DMEM (which does not contain asparagine) for the indicated time points. As a control, cells were treated with tunicamycin (TM, 1 µg/ml) for 8 h. Q: glutamine. Representative example (**B**) of 3 biological replicates quantified in (**C**). The same samples were loaded on different gels and blotted in parallel with the indicated antibodies. Protein concentration was controlled by blotting α-tubulin on a separate gel. Glutamine- and asparagine-rich condition for each genotype is set to 1. Line: average, error bars: standard deviation. (**D**) mTORC1 activity upon glutamine depletion in control or GCN2 KO cells treated with ISRIB. Immunoblot from control or GCN2 KO HEK293T cells treated with vehicle (DMSO) or 200 nM ISRIB and incubated in regular or glutamine-free DMEM for the indicated time points. Q: glutamine. Representative example of 3 biological replicates. The same samples were loaded on different gels and blotted in parallel with the indicated antibodies. Protein concentration was controlled by blotting α-tubulin on a separate gel. (**E**, **F**) Acute mTORC1 inhibition upon glutamine removal does not require eIF2α phosphorylation. Immunoblot from control (eIF2α$^{S51S/S}$) or knock-in MEF cells harboring a mutation that abolishes eIF2a phosphorylation (eIF2α$^{S51A/A}$). Cells were incubated in regular or glutamine-free DMEM for the indicated time points. As a control, cells were treated with amino acid-free DMEM for 8 h. Q: glutamine. Representative example (**E**) of 3 biological replicates quantified in (**F**). The same samples were loaded on different gels and blotted in parallel with the indicated antibodies. Protein concentration was controlled by blotting α-tubulin on a separate gel. Glutamine-rich condition for each genotype is set to 1. Line: average, error bars: standard deviation. (**G**, **H**) Induction of the ISR by tunicamycin does not inhibit mTORC1 acutely. Immunoblot from control HEK293T cells incubated in regular or glutamine-free DMEM for the indicated time points, or treated with vehicle (DMSO) or tunicamycin (TM, 1 µl/ml) for the indicated time points. Q: glutamine. Representative example (**G**) of 3 biological replicates quantified in (**H**). The same samples were loaded on different gels and blotted in parallel with the indicated antibodies. Protein concentration was controlled by blotting α-tubulin on a separate gel. 0 h treatment conditions are set to 1. Line: average, error bars: standard deviation.

© The Author(s)

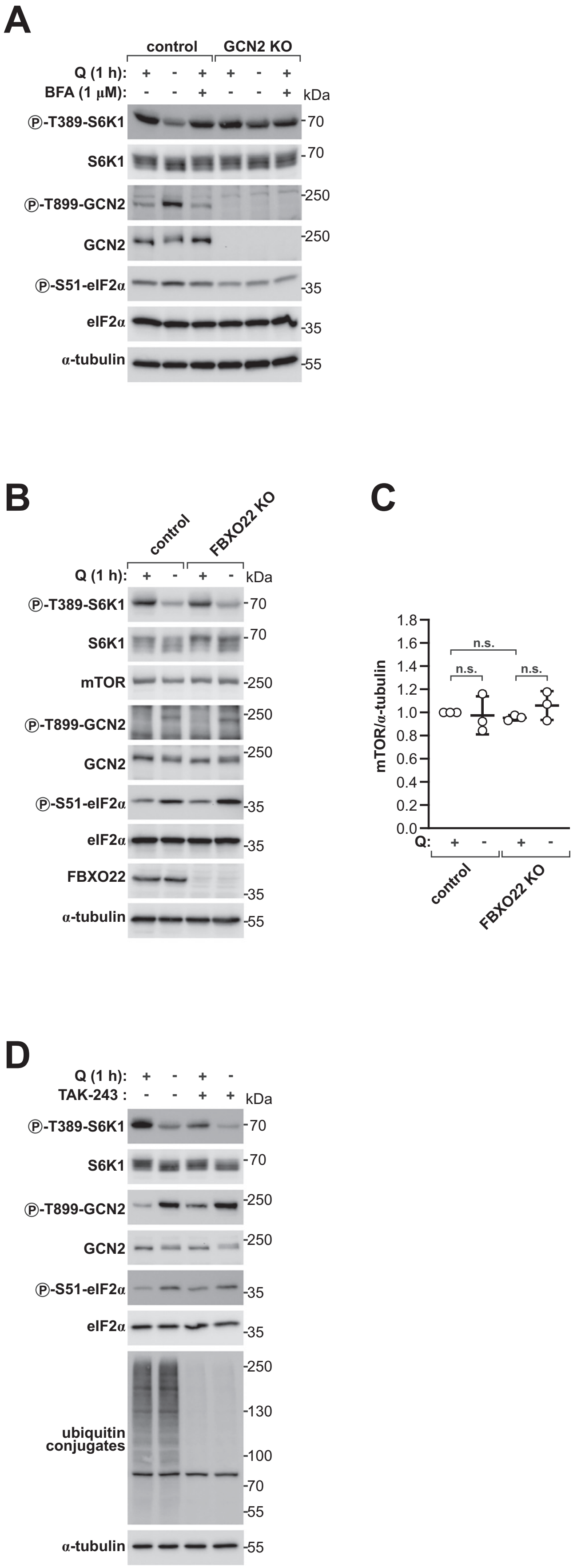
A
control
GCN2 KO
Q (1 h): + - + + - +
BFA (1 μM): - - + - - +
kDa
Ⓟ-T389-S6K1
S6K1
Ⓟ-T899-GCN2
GCN2
Ⓟ-S51-eIF2α
eIF2α
α-tubulin
70
70
250
250
35
35
55
B
control
FBXO22 KO
Q (1 h): + - + -
kDa
Ⓟ-T389-S6K1
S6K1
mTOR
Ⓟ-T899-GCN2
GCN2
Ⓟ-S51-eIF2α
eIF2α
FBXO22
α-tubulin
C
mTOR/α-tubulin
n.s.
n.s.
n.s.
1.8
1.6
1.4
1.2
1.0
0.8
0.6
0.4
0.2
0.0
Q: + - + -
control
FBXO22 KO
D
Q (1 h): + - + -
TAK-243 : - - + +
kDa
Ⓟ-T389-S6K1
S6K1
Ⓟ-T899-GCN2
GCN2
Ⓟ-S51-eIF2α
eIF2α
ubiquitin conjugates
α-tubulin
250
130
100
70
55

 © The Author(s)

Figure EV8. Neither ARF1 nor FBXO22 are involved in mTORC1 inhibition upon acute glutamine starvation.

(**A**) ARF1 inhibition does not affect mTORC1 activity. Immunoblot from control or GCN2 KO HEK293T cells treated with vehicle (DMSO) or 1 μM Brefeldin A (BFA) for 1 h or starved of glutamine in glutamine-free DMEM for 1 h. Q: glutamine. Representative of 3 biological replicates. The same samples were loaded on different gels and blotted in parallel with the indicated antibodies. Protein concentration was controlled by blotting α-tubulin on a separate gel. (**B**, **C**) Inhibition of mTORC1 in response to acute glutamine removal for 1 h does not require FBXO22. Immunoblot from control or FBXO22 KO HEK293T cells incubated in regular or glutamine-free DMEM for 1 h. Q: glutamine. Representative example (**B**) of 3 biological replicates quantified in (**C**). The same samples were loaded on different gels and blotted in parallel with the indicated antibodies. Protein concentration was controlled by blotting α-tubulin on a separate gel. mTOR levels in control unstarved cells are set to 1. Q: glutamine, line: average, error bars: standard deviation. One-way ANOVA and Tukey's post hoc test. n.s.: non significant. (**D**) Inhibition of mTORC1 in response to glutamine removal for 1 h does not require any protein ubiquitination. Immunoblot from control HEK293T cells treated with vehicle (DMSO) or the ubiquitin activating enzyme (UAE) inhibitor TAK-243 (1 μM) and incubated in regular or glutamine-free DMEM for 1 h. Q: glutamine. Representative of 3 biological replicates. The same samples were loaded on different gels and blotted in parallel with the indicated antibodies. Protein concentration was controlled by blotting α-tubulin on a separate gel.

© The Author(s)

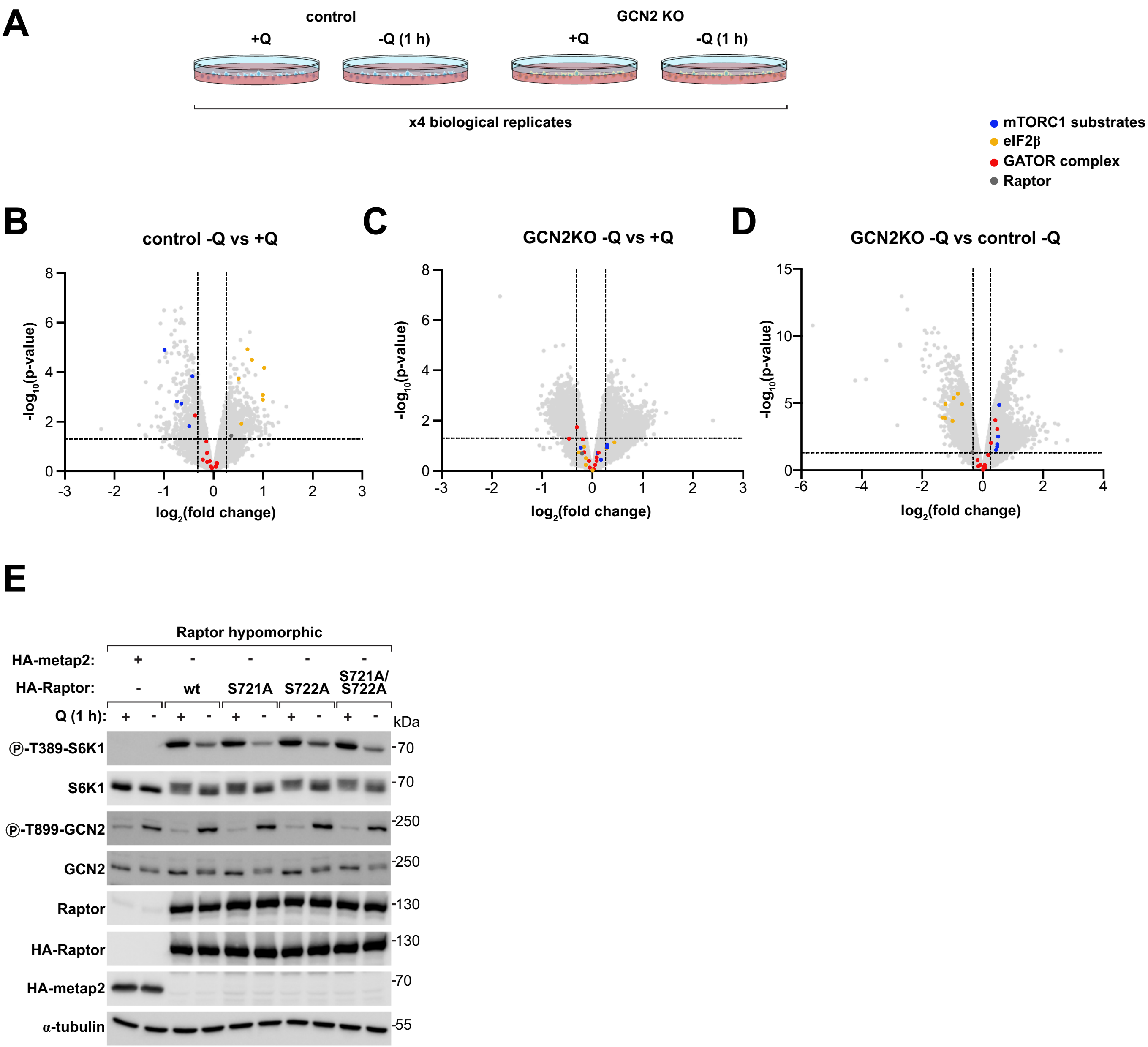

Figure EV9. The GATOR-Rag machinery is not a direct substrate of GCN2.

(**A–D**) Phosphoproteomics analysis of control HEK293T and GCN2 KO cells incubated in regular and glutamine-free DMEM for 1 h, as shown in the scheme in (**A**). (**B–D**) Volcano plots comparing the abundance of phosphorylated peptides between the indicated conditions. eIF2β: phospho-eIF2β (S105, T111, S105/T111). mTORC1 substrates: phospho-4EBP1 (S65/T68, S65/T70, T68/T70), phospho-4EBP2 (S65/T70), phospho-S6 (S235/S236). GATOR complex: phospho-DEPDC5 (S503, S1530, S445), phospho-Mios (S766), phospho-Wdr24 (S594/S598), phospho-Wdr59 (S564, S603), phospho-Szt2 (S719, S1642, S1644/1645, S1651, S1656/S1657). Raptor: phospho-Raptor (S722). Dashed lines correspond to $p = 0.05$ (x axis) and ±1.2 fold change (y axis), $n = 4$ biological replicates. Q: glutamine. (**E**) Inhibition of mTORC1 upon 1 h of glutamine removal does not require phosphorylation of Raptor at Ser721 or Ser722. Immunoblot from control HEK293T cells or Raptor-hypomorphic HEK293T cells that are almost completely lacking Raptor expression and thereby have very low mTORC1 activity. Cells were stably transfected with a HA-tagged control protein (HA-metap2), HA-tagged wild-type Raptor or alanine point mutants abolishing the phosphorylations at S721 and S722, either individually or combined. Cells were incubated in regular or glutamine-free DMEM for 1 h. Q: glutamine. Representative of 3 biological replicates. The same samples were loaded on different gels and blotted in parallel with the indicated antibodies. Protein concentration was controlled by blotting α-tubulin on a separate gel.

 © The Author(s)

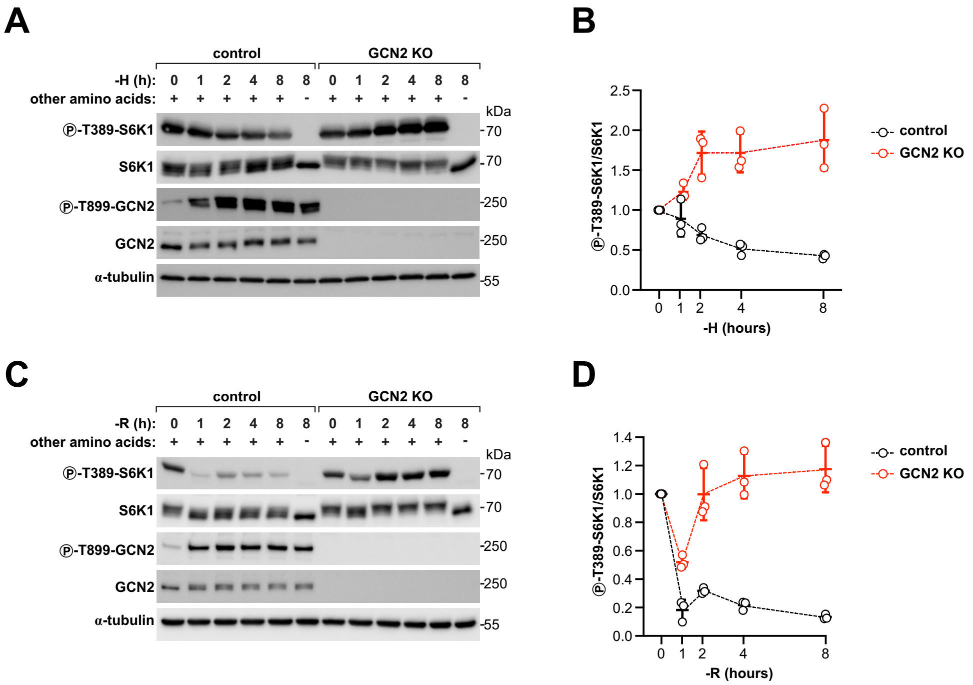

Figure EV10. Response of mTORC1 to arginine or histidine deprivation.

(**A**, **B**) Histidine deprivation causes progressive mTORC1 inhibition in an entirely GCN2-dependent manner. Immunoblot from control or GCN2 KO HEK293T cells incubated in regular or histidine-free DMEM for the indicated time points. As a control, cells were incubated in amino acid-free DMEM for 8 h. H: arginine. Representative example (**A**) of 3 biological replicates quantified in (**B**). The same samples were loaded on different gels and blotted in parallel with the indicated antibodies. Protein concentration was controlled by blotting α-tubulin on a separate gel. Histidine-replete condition for each genotype is set to 1. Line: average, error bars: standard deviation. (**C**, **D**) GCN2 is required for complete and persistent mTORC1 inhibition upon arginine deprivation. Immunoblot from control or GCN2 KO HEK293T cells incubated in regular or arginine-free DMEM for the indicated time points. As a control, cells were incubated in amino acid-free DMEM for 8 h. R: arginine. Representative example (**C**) of 3 biological replicates quantified in (**D**). The same samples were loaded on different gels and blotted in parallel with the indicated antibodies. Protein concentration was controlled by blotting α-tubulin on a separate gel. Arginine-replete condition for each genotype is set to 1. Line: average, error bars: standard deviation.

© The Author(s)

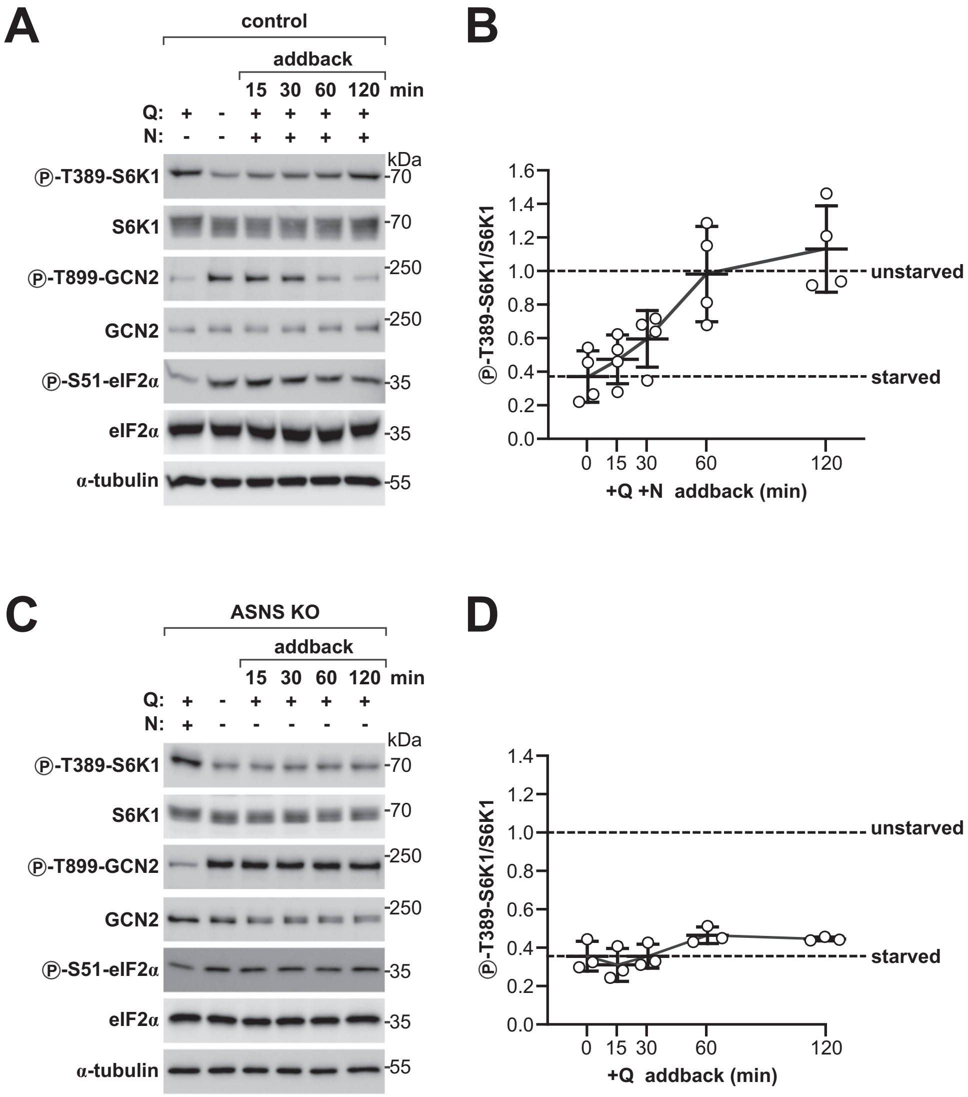

Figure EV11. Metabolism to asparagine is required for mTORC1 reactivation upon addback of glutamine.

(**A**, **B**) mTORC1 is reactivated slowly upon glutamine addback also when asparagine is additionally supplemented. Immunoblot from control HEK293T cells treated with glutamine removal and re-addition of glutamine together with 25 µM asparagine for the indicated time points. Q: glutamine, N: asparagine. Representative examples (**A**) of 3 biological replicates quantified in (**B**). The same samples were loaded on different gels and blotted in parallel with the indicated antibodies. Protein concentration was controlled by blotting α-tubulin on a separate gel. Unstarved cells are set to 1. Line: average, error bars: standard deviation. (**C**, **D**) mTORC1 reactivation upon glutamine addback requires its metabolism to asparagine. ASNS KO HEK293T cells were incubated in regular DMEM supplemented with 25 µM asparagine for 1 h, or starved of glutamine- and asparagine for 1 h and then re-stimulated with glutamine for the indicated time points. Q: glutamine, N: asparagine. Representative example (**C**) of 3 biological replicates quantified in (**D**). The same samples were loaded on different gels and blotted in parallel with the indicated antibodies. Protein concentration was controlled by blotting α-tubulin on a separate gel. Unstarved cells are set to 1. Line: average, error bars: standard deviation.

 © The Author(s)
